# Exploiting the Chalcone Scaffold to Develop Multifunctional Agents for Alzheimer’s Disease

**DOI:** 10.3390/molecules23081902

**Published:** 2018-07-30

**Authors:** Angela Rampa, Manuela Bartolini, Letizia Pruccoli, Marina Naldi, Isabel Iriepa, Ignacio Moraleda, Federica Belluti, Silvia Gobbi, Andrea Tarozzi, Alessandra Bisi

**Affiliations:** 1 Department of Pharmacy and Biotechnology, Alma Mater Studiorum-University of Bologna, Via Belmeloro 6, 40126 Bologna, Italy; manuela.bartolini3@unibo.it (M.B.); marina.naldi@unibo.it (M.N.); federica.belluti@unibo.it (F.B.); silvia.gobbi@unibo.it (S.G.); 2 Department for Life Quality Studies, Alma Mater Studiorum-University of Bologna, Corso d’Augusto 237, 47921 Rimini, Italy; letizia.pruccoli2@unibo.it (L.P.); andrea.tarozzi@unibo.it (A.T.); 3 Department of Organic Chemistry and Inorganic Chemistry, School of Sciences, University of Alcalá, E-28871 Alcalá de Henares, Madrid, Spain; isabel.iriepa@uah.es (I.I.); ignacio.moraleda@uah.es (I.M.)

**Keywords:** Alzheimer’s disease, chalcones, cholinesterase inhibitors, antioxidants, neuroprotection

## Abstract

Alzheimer’s disease still represents an untreated multifaceted pathology, and drugs able to stop or reverse its progression are urgently needed. In this paper, a series of naturally inspired chalcone-based derivatives was designed as structural simplification of our previously reported benzofuran lead compound, aiming at targeting both acetyl (AChE)- and butyryl (BuChE) cholinesterases that, despite having been studied for years, still deserve considerable attention. In addition, the new compounds could also modulate different pathways involved in disease progression, due to the peculiar *trans*-α,β-unsaturated ketone in the chalcone framework. All molecules presented in this study were evaluated for cholinesterase inhibition on the human enzymes and for antioxidant and neuroprotective activities on a SH-SY5Y cell line. The results proved that almost all the new compounds were low micromolar inhibitors, showing different selectivity depending on the appended substituent; some of them were also effective antioxidant and neuroprotective agents. In particular, compound **4**, endowed with dual AChE/BuChE inhibitory activity, was able to decrease ROS formation and increase GSH levels, resulting in enhanced antioxidant endogenous defense. Moreover, this compound also proved to counteract the neurotoxicity elicited by Aβ_1–42_ oligomers, showing a promising neuroprotective potential.

## 1. Introduction

Alzheimer’s disease (AD) is the most common cause of dementia and is becoming a global health concern. Indeed, the continuous rise in the aging population and an exponential increase in AD cases over the next few decades in Europe are expected to result in immense pressure on the social and health-care systems of the region [1,2]. Therefore, there is an urgent need for developing curative or disease-modifying therapies to offset the upcoming AD epidemic. However, despite millions of dollars of investment in drug discovery and clinical trials, no single molecule has yet been approved for its treatment since the advent of cholinesterase inhibitors (ChEIs) and memantine, an *N*-methyl-d-aspartate (NMDA) receptor antagonist [3].

In AD, atrophy of cells’ nuclei in the key areas of the brain, such as the basal forebrain, leads to a noticeable decrease in cholinergic function; cholinergic transmission in the brain is an essential component of cognitive function (cholinergic hypothesis). For this reason, drug therapies have been developed focusing on ChEIs, which increase cholinergic transmission through the inhibition of the enzymes responsible for breaking down the neurotransmitter acetylcholine. ChEIs are currently used for the symptomatic treatment of AD, since they may offer some stabilization of the symptoms, which ultimately could provide patients with better quality of life in their remaining years, even if they do not alter the underlying disease trajectory [4].

Furthermore, AD is characterized by misfolded protein aggregates [5], chronic inflammation [6] and oxidative stress, elicited by increased generation of reactive oxygen species (ROS) [7]. Oxidative stress merely reflects a disequilibrium between the amount of oxidants produced and the quantities of antioxidant enzymes—such as superoxide dismutases, catalases and glutathione peroxidase—required to restore the appropriate balance. This imbalance has its origins in genes and the ways in which gene expression is regulated: a central role is played by the transcription factor called nuclear factor (erythroid-derived 2)-like 2 or Nrf2, the “master regulator” of the antioxidant response that modulates the expression of hundreds of genes, including antioxidant enzymes [8]. Young healthy individuals possess all the genetic resources required to maintain a correct oxidative balance, whereas Nrf2 expression appears to decline with aging, leading to a dysregulation of oxidative stress responses. Accordingly, the use of Nrf2 activators has been attracting increasing attention as a valuable therapeutic strategy for neurodegenerative diseases. In particular, cellular Nrf2 levels are tightly regulated by Keap1, a protein acting as an oxidative stress sensor through modifications of various cysteine residues located in different protein regions. These modifications lead to conformational changes that disrupt the Keap1/Nrf2 interaction, inhibiting the degradation of Nrf2 and enabling its binding to antioxidant response elements (ARE) and the following activation of antioxidant enzymes transcription [8].

**Figure 1:**
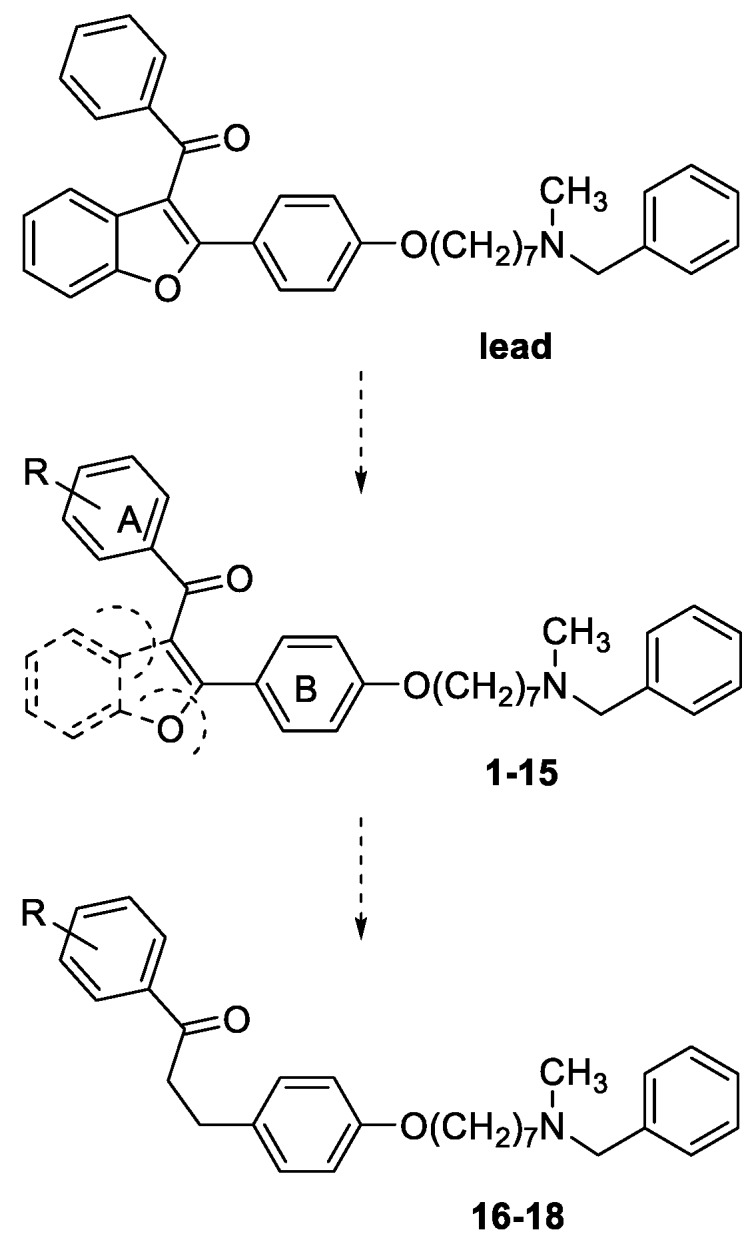
Design strategy for the studied compounds **1**–**18**.

Our research group has been involved for many years in the development of drug candidates for AD treatment. In particular, in previous papers we reported a promising benzofuran-based lead compound endowed with multiple biologic properties (Figure 1) [9,10]. Here we report the design and evaluation of chalcone-based compounds and the corresponding hydrogenated derivatives as flexible open ring analogs of this lead. Chalcones (*trans*-1,3-diaryl-2-propen-1-ones), found as central core in a variety of important bioactive compounds [11], belong to the flavonoid family, the largest class of secondary metabolites in plants, where they serve as a defense mechanism to counteract ROS in order to survive and prevent molecular damage. These natural products can be defined as “privileged structures” and they can be properly exploited to develop effective strategies in different fields of drug discovery [12]. Indeed, chalcones possess a peculiar *trans*-α,β-unsaturated ketone, a functional group perceived as a potential Michael acceptor, that allows the molecule to interact with cysteine residues of different biological targets to obtain the Michael adduct [12,13,14].

The simpler and more flexible framework of the new chalcone-based derivatives allowed the obtaining of less constrained molecules, which could better interact with the narrow gorge of the ChE enzymes. In this new series, the A ring was decorated with a methoxy group or an additional tertiary amino function, charged at physiological pH and linked through spacers of different lengths, to evaluate its possible binding to the peripheral anionic site (PAS) located at the top of the acetylcholinesterase (AChE) gorge. To better address this point, the amino side chain was introduced in *meta* or *para* positions on the benzene ring, as in compounds **1**–**15**. Finally, to assess the role of the chalcone α,β-double bond and to evaluate the impact of a further increase in flexibility, the saturated analogs **16**–**18** were also designed and synthesized. The new compounds were reported in Table 1 and Table 2.

## 2. Results and Discussion

### 2.1. Chemistry

**Scheme 1:**
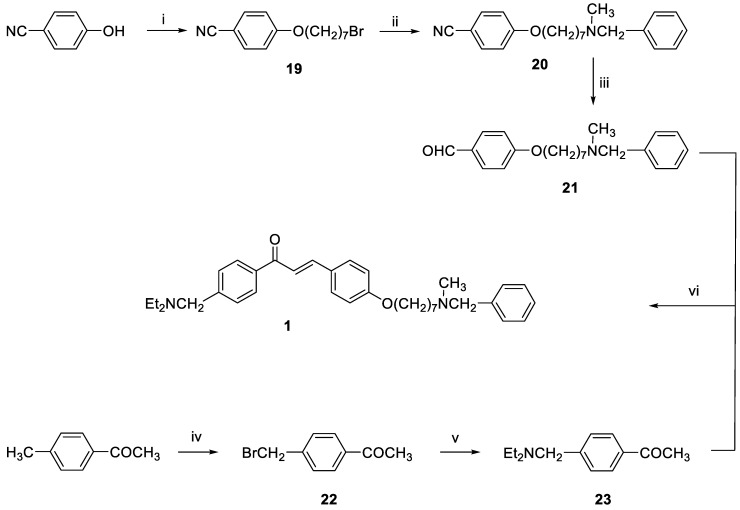
Reagents and conditions: (i) Br(CH_2_)_7_Br, K_2_CO_3_, acetone, reflux; (ii) *N*-methylbenzylamine, Et_3_N, toluene, reflux; (iii) Ni/Raney, HCOOH, reflux; (iv) NBS, (PhCOO)_2_, CCl_4_, reflux; (v) Et_2_NH, toluene, reflux; (vi) KOH, EtOH, r.t.

According to Scheme 1, compound **1** was synthesized starting from 4-hydroxybenzonitrile, which was alkylated, in the presence of K_2_CO_3_, with 1,7-dibromoheptane obtaining **19**, that was then subjected to nucleophilic substitution reaction by *N*-methylbenzylamine to afford **20**. Afterwards, the cyano group was reduced using the Ni/Raney alloy in formic acid [15] to give the aldehyde **21**. Benzylic bromination with *N*-bromosuccinimide (NBS) of 4-methylacetophenone afforded **22**, which was subsequently reacted with diethylamine to give **23**. Chalcone **1** was obtained via Claisen-Schmidt condensation reaction of **23** with the previously prepared aldehyde **21**.

**Scheme 2:**
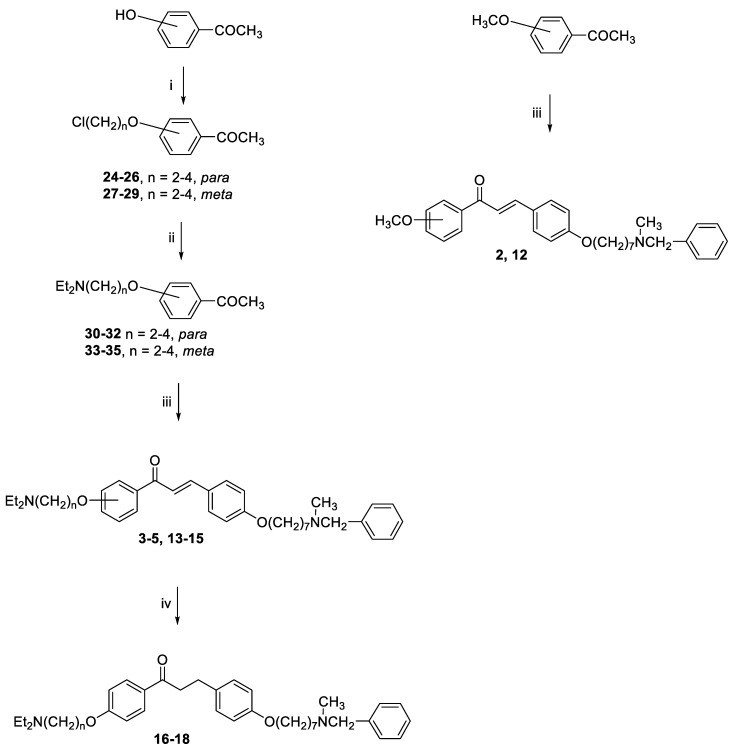
Reagents and conditions: (i) Cl(CH_2_)_n_Br, K_2_CO_3_, acetone, reflux; (ii) selected amine, toluene, reflux; (iii) **21**, KOH, EtOH, r.t.; (iv) H_2_, Pd/CaCO_3_, THF, r.t.

The synthesis of compounds **2**–**18** were accomplished as shown in Scheme 2. The 3- or 4-hydroxyacetophenone was alkylated, in the presence of K_2_CO_3_, with the selected bromochloroalkane to afford the chloroalkyloxy derivatives **24**–**29**, which were then subjected to nucleophilic substitution by diethylamine in refluxing toluene to give compounds **30**–**35**. The chalcones **3**–**5** and **13**–**15** were obtained via Claisen-Schmidt condensation of **30**–**35** with the previously described aldehyde **21** (see Scheme 1). With the same procedure (Scheme 2, right side), compounds **2** and **12** were obtained starting from the selected methoxyacetophenone and **21**. Finally, compounds **3**–**5** were hydrogenated with Pd/CaCO_3_ to obtain the final derivatives **16**–**18**.

**Scheme 3:**
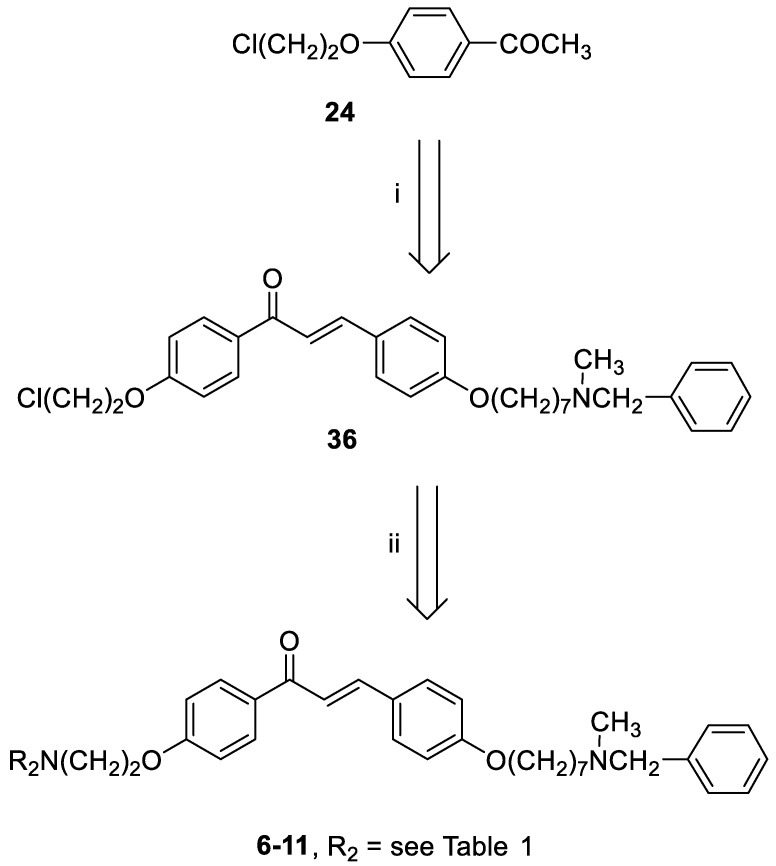
Reagents and conditions: (i) **21**, KOH, EtOH, r.t.; (ii) selected amine, toluene, reflux.

The derivatives bearing amines other than diethylamine were prepared according to Scheme 3: **24** was first subjected to Claisen-Schmidt condensation reaction with **21** to give chalcone **36** that, via a parallel synthesis procedure, was then subjected to nucleophilic substitution reaction by the selected amine (**6**–**11**).

### 2.2. Inhibition of Cholinesterase Activity

The inhibitory potencies of compounds **1**–**18** toward human recombinant AChE (*h*AChE) and BuChE from human serum (*h*BuChE) were determined using Ellman’s assay [16] and the IC_50_ values were reported in Table 1 and Table 2.

Regarding *h*AChE, the introduction of a methoxy group on the chalcone A ring, independently of its *para* or *meta* position, as in compounds **2** and **12**, respectively, led to a consistent drop in potency with respect to the benzofuran-based lead compound (about 100 µM vs 40.7 µM [9], Table 1). The substitution of the methoxy with a methylendiethylamino group, in compound **1**, induced a 35-fold increase in inhibitory activity (2.81 µM), leading us to speculate on a positive contribution of the diethylamino group. An additional structural modification, namely the lengthening of the side chain from 2 to 4 methylene units, combined with the introduction of an oxygen atom, allowed an increase in activity of one order of magnitude, as in the *para*-alkoxymethylendiethylamino derivatives **3**–**5**, showing potency in the sub-micromolar range. In this context, the *meta*-substitution seemed to negatively affect activity, compounds **13**–**15** being less effective as AChE inhibitors than the corresponding *para*-substituted compounds. In this small *meta*- subset, compound **15**, with 4-methylene units, was the most active.

One of the aims of this paper was the design of structurally simpler and more flexible derivatives with respect to our previously reported benzofuran-based lead compound, in order to exploit this increased adaptability in a better interaction with AChE. The results indicate that the introduction of a *para*-diethylaminoethoxy group (compound **3**) led to a 10-fold increase in potency. Moreover, a positive contribution of the oxygen atom in the side chain can be recognized, maybe due to its H-bond acceptor feature, since compound **1**, bearing a simple diethylaminomethyl group, proved to be less active.

Taking this into account, for a deeper evaluation of the contribution of the terminal amino group in the interaction with PAS, the ethoxy spacer was maintained and diethylamine was replaced with different bulkier amines (Table 1).

Surprisingly, among the selected amines, only piperidine (**7**) enabled the compound to retain a micromolar inhibitory potency, whereas all the other amino functions led to significantly less active compounds (**6**, **8**–**11**). In particular, the morpholino derivative (**6**) was about 500-fold worse than **7**. In order to explain this unexpected result, docking studies were performed (see below).

Finally, the α,β unsaturation of derivatives **3**–**5** was selectively hydrogenated, aiming at evaluating its role in the orientation of the molecules. This modification, leading to the more flexible compounds **16**–**18** (Table 2), allowed maintaining a micromolar inhibitory potency, even if lower with respect to **3**–**5**.

Regarding *h*BuChE, the introduction of a methoxy group on the A ring, both in *para* and in *meta* positions, as in compounds **2** and **12**, led to a consistent increase in potency with respect to AChE (3.42 µM vs. 98.5 µM for **2** and 8.58 µM vs. 105 µM for **12**, Table 1). A similar trend was observed for the benzofuran-based lead compound (3.42 and 8.58 µM, respectively, and 38.1 µM [9]).

Differently from what was noticed for *h*AChE inhibition, the substitution of the methoxy with a diethylamino group, as in compounds **1**, **4** and **5**, allowed maintaining of the activity in the micromolar range, although with different selectivity depending on the position of the chain. Indeed, the subset bearing the diethylamino group in *para* position showed a greater affinity for *h*AChE, while the *meta* substituted one proved to be more potent on *h*BuChE. In this small subset of *meta*-derivatives, compound **13**, with 2-methylene units, was the most active and the most potent of the whole series on this enzyme.

As expected, the larger size of the active site gorge in BuChE, due to the presence of the smaller valine and leucine instead of the aromatic phenylanine residues [17], enabled a better fit of molecules with bulky substituents, with the exception of compounds **10** and **11**, which proved to be inactive on both ChEs. 

To complete the picture, the hydrogenated compounds **16**–**18** (Table 2) were three/four-fold less potent on BuChE with respect to AChE.

In a healthy brain, AChE is the major player in the breakdown of the neurotransmitter acetylcholine (ACh). Conversely, in an AD affected brain, AChE levels progressively decrease, while BuChE increases and becomes prevailing [18]. As a result, the regulation of ACh central levels in AD patients likely involves both enzymes, which thus represent validated therapeutic targets to counteract the cholinergic deficit [19,20]. Moreover, recent studies indicate that BuChE can be associated with Aβ plaques, due to its accumulation in these structures, and inhibition of this enzyme could be responsible for a reduced Aβ deposition in subcortical regions of the brain [21]. In this overall framework, compound **4** emerges as the most promising, being an effective low micromolar inhibitor of both enzymes.

### 2.3. Docking Studies

**Figure 2:**
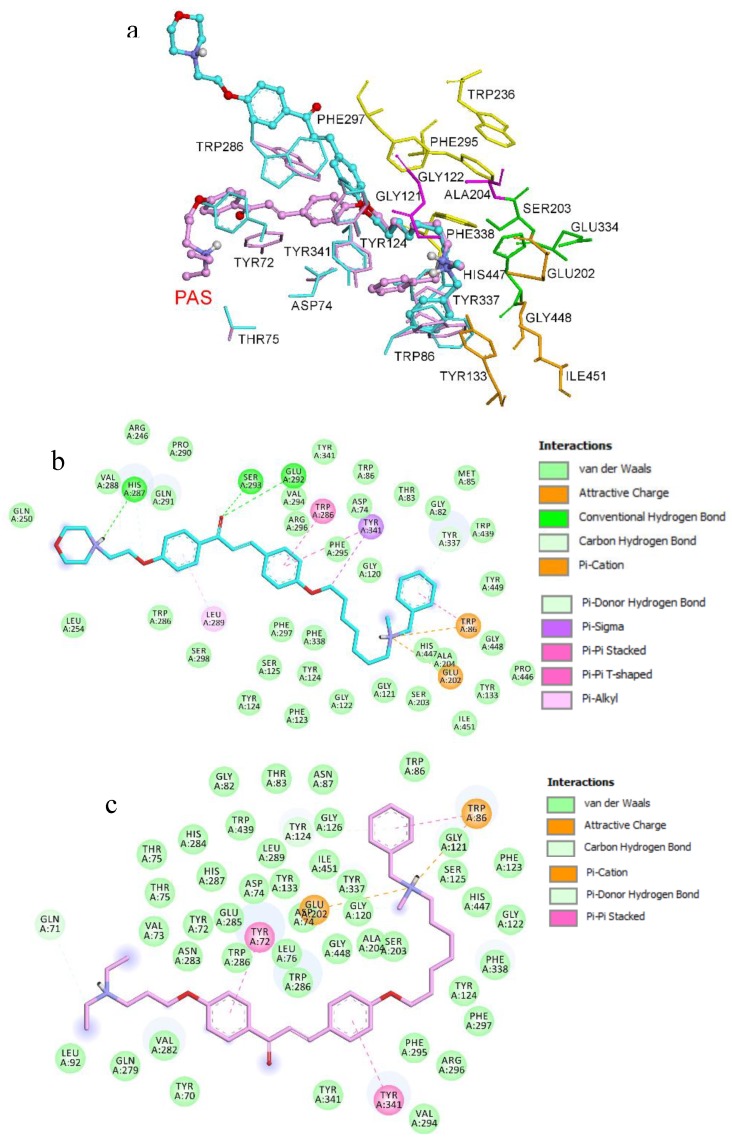
Docked pose of inhibitors **4** and **6** at the active site of *h*AChE. (**a**) Compounds **4** and **6** are rendered as ball and sticks (**4**, carbon atoms in pink; **6**, carbon atoms in blue) and the side chain conformations of the mobile residues are illustrated in pink and blue, respectively. The catalytic triad (CT) is colored in green, the oxyanion hole (OH) in magenta, the anionic sub-site (AS) in orange, except Trp86, the acyl binding pocket (ABP) in yellow and PAS in light pink and blue; (**b**) 2D-representation of interactions established by **4** with *h*AChE; (**c**) 2D-representation of interactions established by **6** with *h*AChE.

To explain the binding mode and the affinities of newly synthesized compounds to target enzymes AChE and BuChE, docking studies were performed and interactions of compounds **4** and **6** with AChE and BuChE were evaluated. Three-dimensional (3D) and two-dimensional (2D) representations of the most energetically profitable poses of these compounds docked in the active site of *h*AChE are presented in Figure 2.

The most energetically favorable binding mode of compound **4** revealed that the *N*-benzylmethylamine moiety binds to the CAS while the diethylamino group is located in the pocket forming PAS and the linker occupied the center of both cavities (Figure 2a). The AChE-**4** complex is stabilized by π-cation and π-π stacking interactions (Figure 2b).

Analysis of the optimal binding mode for compound **6** places the ligand within the active site of AChE. The simulations showed a clear preference to accommodate the *N*-benzylmethylamine moiety within the CAS while the morpholine moiety is turned away from the PAS. The key factors to stabilize the enzyme-ligand complex were found to be π-cation, π–π T-shaped, π–π stacking and hydrogen bonding interactions (Figure 2c).

**Figure 3:**
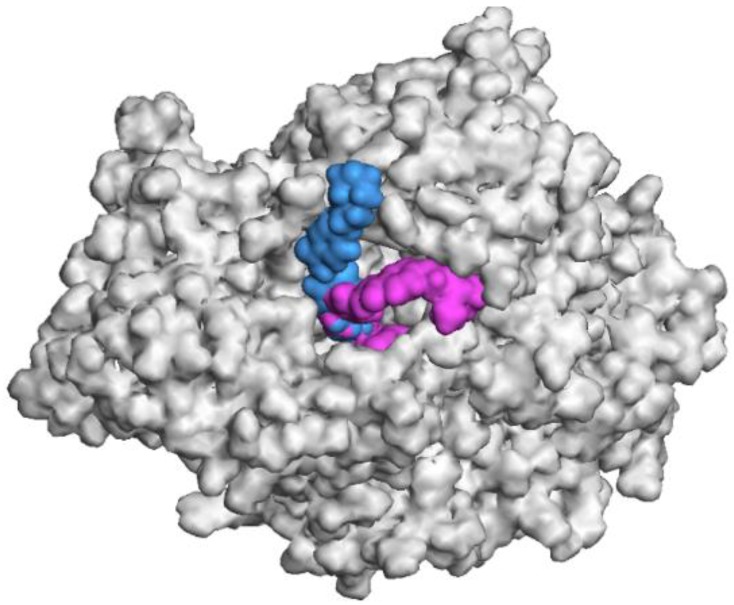
3D surface representation of compounds **4** (pink), **6** (blue) and *h*AChE (white).

Figure 3 illustrates the docked pose of compound **4** superimposed with the docked pose of compound **6**, pointing out to the fact that these two molecules do not share a similar binding mode. Both compounds **4** and **6** could interact with the catalytic active site with Trp86 and Glu202 residues; however, compound **4** also interacted in the peripheral anionic site. The higher binding of compound **4** within AChE binding pocket can be attributed to the binding orientation and geometry permitted by this ligand, spanning both cavities of AChE (CAS and PAS).

**Figure 4:**
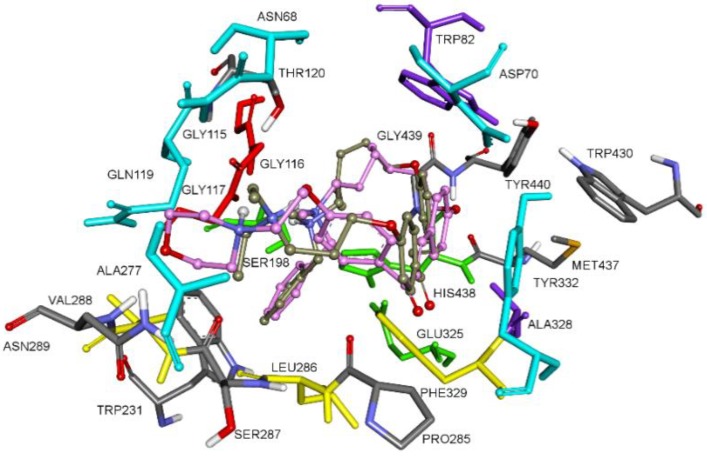
Proposed binding mode for compounds 4 and 6 inside the gorge cavity of *h*BuChE. Compound **4** is colored olive green. Compound **6** is colored pink. Different subsites of the active site were colored: catalytically anionic site (CAS) in green, oxyanion hole (OH) in red, choline binding site in violet (CBS), acyl binding pocket (ABP) in yellow, and peripheral site (PAS) in blue.

Compounds **4** and **6** were modeled into the structure of *h*BuChE (PDB: 4BDS, Figure 4). Both ligands fit into the active site of *h*BuChE mainly through π–π and hydrogen bonds interactions, which are found to be essential for binding. Thus, compound **4** interacts inside the *h*BuChE binding cavity in the same region occupied by compound **6**. These compounds find interactions in the middle of the active-site gorge and the *N*-benzylmethylamine moiety is pointed toward the catalytic triad residues, His438, Ser198 and Glu325.

**Figure 5:**
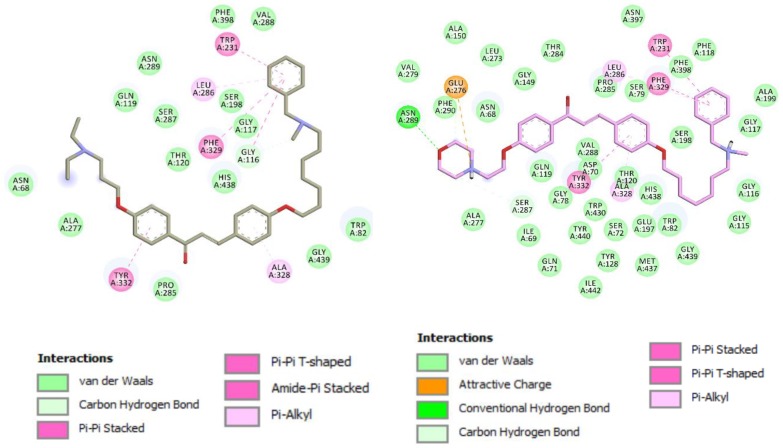
2D Schematic representation of different interactions of compounds **4** (**left**) and **6** with *h*BuChE (**right**).

As shown in Figure 5, it was found that the *N*-benzylmethylamine moiety of both ligands occupied the catalytic site of *h*BuChE through π–π stacking interactions with Trp231 and Phe329. Furthermore, the binding in compound **6** is also supported by the formation of a hydrogen bond between a morpholine oxygen atom of the ligand and Asn289. In addition, the other ammonium groups are located at the beginning of the gorge. These ligands bind to *h*AChE with extended conformations, whereas they bind to *h*BuChE with folded conformations

### 2.4. Antioxidant Activity

To deeply investigate the biological profile of the new derivatives, a small subset of compounds was evaluated to establish their antioxidant potential, which could be associated to the chalcone scaffold. To this aim, derivatives **2**, **4** and **17** were selected, due to their different structural features: **2** possesses a simple methoxy group, **4** is a dual AChE/BuChE inhibitor bearing an amino terminal group and **17** is devoid of the chalcone peculiar double bond.

To establish the range of concentrations not associated with neurotoxicity, SH-SY5Y cells viability was evaluated after a 24 h treatment with compounds **2**, **4** and **17** at different concentrations (2.5–80 µM) using the MTT assay. The obtained results highlighted that concentrations up to 2.5 µM did not affect neuronal viability (data not shown). Therefore, to perform the subsequent antioxidant and neuroprotective assays, a concentration of 1.25 µM was selected for all the studied compounds.

**Figure 6:**
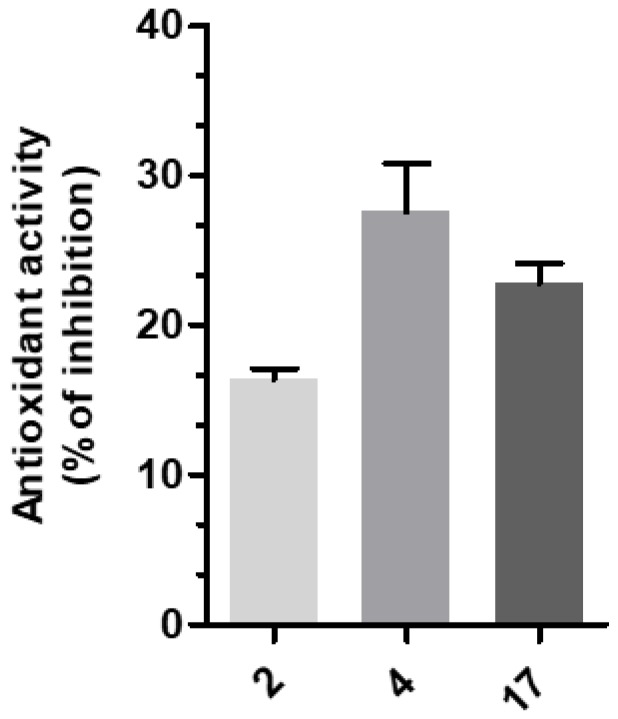
Antioxidant activity of compounds 2, 4 and 17 against the ROS formation induced by *t*-BuOOH in SH-SY5Y cells. Cells were incubated with compounds **2**, **4** and **17** (1.25 µM) for 24 h and then treated with *t*-BuOOH (100 µM) for 30 min. At the end of incubation, intracellular ROS formation was detected using the fluorescent probe H_2_DCF-DA, as described in the Materials and Methods section. Data are expressed as antioxidant activity in terms of inhibition percentage in ROS formation induced by *t*-BuOOH and are reported as mean ± SEM of three independent experiments.

To determine the antioxidant activity, the intracellular ROS formation induced by *t*-BuOOH was evaluated after a 24 h treatment with compounds **2**, **4** and **17** (1.25 µM) using the fluorescent probe H_2_DCF-DA. Data showed that the studied compounds prevented ROS formation in the order of strength **4** > **17** > **2**, with inhibition percentages of 27%, 23% and 16%, respectively (Figure 6).

**Figure 7:**
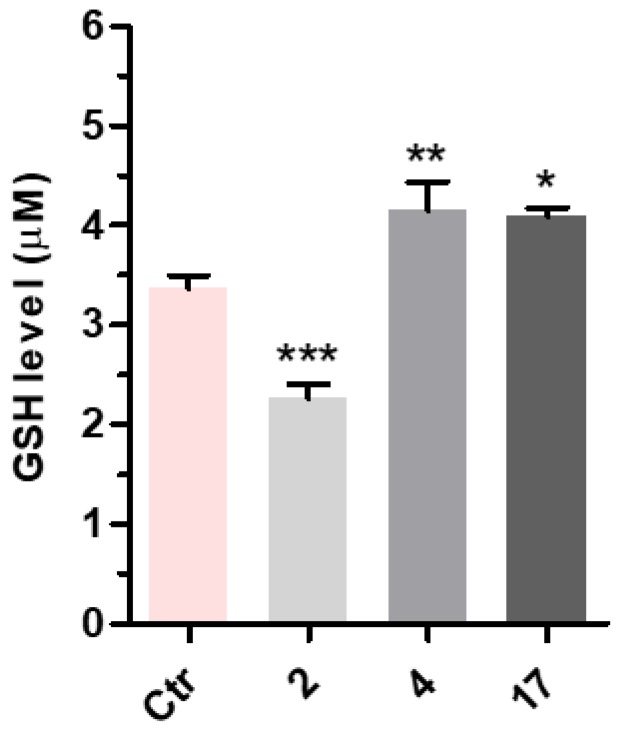
Effects of compounds **2**, **4** and **17** on GSH levels in SH-SY5Y cells. Cells were incubated with compounds **2**, **4** and **17** (1.25 µM) for 24 h. At the end of incubation, GSH levels were detected using the fluorescent probe MCB, as described in the Materials and Methods section. Data are expressed as concentration of GSH (µM) and reported as mean ± SEM of three independent experiments (* *p* < 0.05, ** *p* < 0.01 and *** *p* < 0.001 vs. control at one way ANOVA with Dunnett post hoc test).

In parallel, glutathione (GSH) levels in SH-SY5Y cells were evaluated after a 24 h treatment with compounds **2**, **4** and **17** (1.25 µM) using the fluorescent probe monochlorobimane (MCB). This evaluation was based on experimental evidence indicating that oxidative stress and aging reduced GSH levels. Data showed that compounds **4** and **17** significantly increased GSH levels, while compound **2** decreased GSH basal levels (Figure 7).

### 2.5. Neuroprotective Activity

It is well documented that soluble Aβ oligomers are neurotoxic species, able to trigger cognitive deficits also in the absence of plaques. Thus, they can be considered critical factors in the pathogenesis of AD by causing synaptic dysfunction and neuronal death [22].

**Figure 8:**
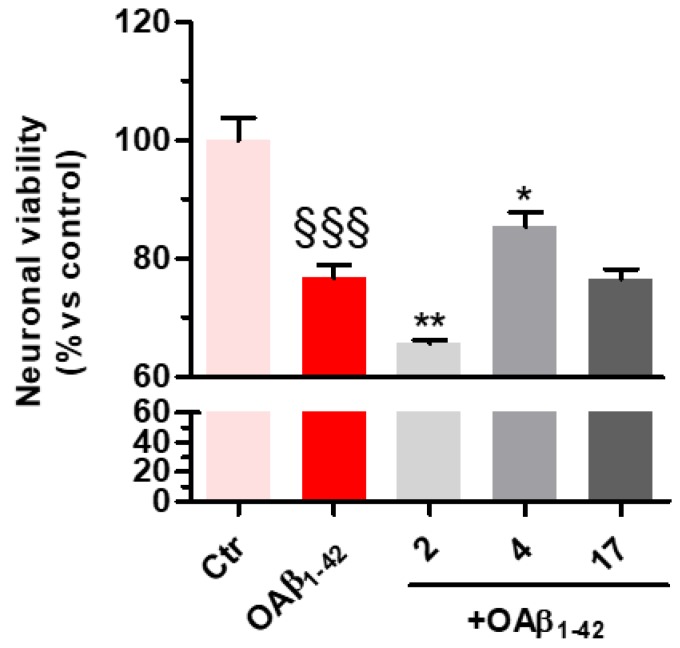
Effects of compounds **2**, **4** and **17** on the neurotoxicity induced by OAβ_1–42_ in SH-SY5Y cells. Cells were incubated with compounds **2**, **4**, **17** (1.25 µM) and OAβ_1–42_ (10 µM) for 4 h. At the end of incubation, the neuroprotective activity of the compounds was detected by the solubilization of intracellular MTT granules in Tween-20, as described in the Materials and Methods section. Data are expressed as percentage of neuronal viability and reported as mean ± SEM of three independent experiments (^§§§^ *p* < 0.001 vs. control, * *p* < 0.05 and ** *p* < 0.01 vs. cells treated with OAβ_1–42_ at one way ANOVA with Bonferroni post hoc test).

The neuroprotective activity toward Aβ_1–42_ oligomers (OAβ_1–42_) (10 µM) induced toxicity in SH-SY5Y cells was evaluated after 4 h treatment with compounds **2**, **4**, **17** (1.25 µM) using the MTT formazan exocytosis assay. As shown in Figure 8, compound **4** partially counteracted the neurotoxic effects induced by OAβ_1–42_ increasing SH-SY5Y cells viability, while compound **2** reinforced the neurotoxic effects induced by OAβ_1–42_. No neuroprotective effect was observed for compound **17**.

Taken together, the data for antioxidant and neuroprotective activities point at compound **4** as the most promising one, being endowed with a better profile than compounds **2** and **17**, suggesting that both the amino terminal group and the chalcone peculiar double bond were crucial structural features for inducing these effects. Undoubtedly, the α,β-unsaturated carbonyl moiety in compound **4**, acting as a Michael acceptor, could interfere with Keap1-Nrf2 binding, causing the subsequent activation of Nrf2 signaling pathway [23]. Indeed, several studies postulate for electrophylic compounds a possible Cys-based modification of Keap1 allowing its dissociation from Nrf2 and leading to the transcription of cytoprotective genes [24,25]. In this respect, a fine-tuning of the chalcone electrophilicity, due to the substituents introduced, can be considered an important feature to minimize the risk of off-target effects. On the other hand, compound **17**, only devoid of the α,β double bond with respect to **4**, could still increase the GSH levels, probably acting with a different mechanism.

In summary, the increased flexibility of these newly synthesized chalcone-based derivatives led to an improved cholinesterase inhibitory activity with respect to the benzofuran lead compound, possibly due to a better fit into the catalytic ChEs gorges. On the other hand, the presence of the distinctive α,β-unsaturated carbonyl moiety also allowed introducing an appreciable antioxidant and neuroprotective potential. In this series, compound **4**, endowed with a dual AChE/BuChE low micromolar inhibitory activity and a relevant antioxidant and neuroprotective profile, emerged as an effective multipotent molecule, suitable to be further developed in view of the multifaceted character of AD.

## 3. Materials and Methods

### 3.1. Chemistry

General Methods. Melting points were measured in glass capillary tubes on a Büchi SMP-20 apparatus and are uncorrected. ^1^H-NMR and ^13^C-NMR spectra were recorded in CDCl_3_, unless otherwise indicated, on a Varian Gemini spectrometer 400 MHz and 101 MHz, respectively. Chemical shifts are reported in parts per million (ppm) relative to tetramethylsilane (TMS), and spin multiplicities are given as s (singlet), d (doublet), t (triplet), m (multiplet) or br (broad). Direct infusion ES-MS spectra were recorded on a Waters Micromass ZQ 4000 apparatus (Waters Alliance, San Diego, CA, USA). Chromatographic separations were performed by flash chromatography on silica gel columns (Kieselgel 40, 0.0400–0.063 mm; Merck, Darmstadt, Germany). Organic solutions were dried over anhydrous sodium sulfate. All chemicals were purchased from Aldrich Chemistry, Milan (Italy), or from Alfa Aesar, Milan (Italy), and were of the highest purity. Compounds were named relying on the naming algorithm developed by CambridgeSoft Corporation (Waltham, MA, USA) and used in ChemDraw Professional 15.0 (PerkinElmer Inc., Waltham, MA, USA).

General method for the synthesis of final compounds **1**–**5**, **12**–**15** and of intermediate **36**: To a mixture of the selected acetophenone (0.001 mol) and **21** (0.001 mol) in EtOH (10 mL), a solution of KOH (1 g, 0.018 mol, in 5 mL of H_2_O) was added, and the reaction mixture was stirred at rt overnight. The mixture was poured into ice and the resulting yellow solid was filtered off. The residue was purified by flash chromatography on silica gel (toluene/acetone 3:2, then MeOH).

*3-(4-((7-(Benzyl(methyl)amino)heptyl)oxy)phenyl)-1-(4-((diethylamino)methyl)phenyl)prop-2-en-1-one* (**1**). Using the previous procedure and starting from **21** and **23**, **1** (yield 55%) was obtained as an oil. ^1^H-NMR δ 1.03 (t, *J* = 7.2 Hz, 6H, 2CH_3_), 1.25–1.61 (m, 8H, 4CH_2_), 1.64–1.85 (m, 2H, CH_2_), 2.19 (s, 3H, NCH_3_), 2.35 (t, *J* = 7.2 Hz, 2H, CH_2_N), 2.42–2.60 (m, 4H, 2CH_2_), 3.46 (s, 2H, CH_2_), 3.62 (s, 2H, NCH_2_), 3.99 (t, *J* = 6.8 Hz, 2H, OCH_2_), 6.86–7.99 (m, 15H, Ar). ^13^C-NMR δ 11.88, 26.08, 27.31, 27.42, 29.22, 29.37, 42.25, 47.05, 57.45, 57.50, 62.32, 68.25, 115.02, 119.81, 127.06, 127.60, 128.31, 128.53, 128.62, 129.00, 129.25, 130.31, 137.30, 139.03, 144.59, 145.49, 161.36, 190.35. MS (ES) *m*/*z*: 527 [M + H]^+^. C_35_H_46_N_2_O_2_. 

*3-(4-((7-(Benzyl(methyl)amino)heptyl)oxy)phenyl)-1-(4-methoxyphenyl)prop-2-en-1-one* (**2**). Using the previous procedure and starting from 4-methoxyacetophenone and **21**, **2** (yield 75%) was obtained as an oil. ^1^H-NMR δ 1.22–1.59 (m, 8H), 1.64–1.82 (m, 2H, CH_2_), 2.20 (s, 3H, NCH_3_), 2.36 (t, *J* = 7.2 Hz, 2H, CH_2_N), 3.48 (s, 2H, CH_2_), 3.88 (s, 3H, OCH_3_), 4.00 (t, *J* = 6.8 Hz, 2H, OCH_2_), 6.88 (m, 3H, Ar), 7.20–7.84 (m, 10H, Ar), 8.02 (d, *J* = 8.8 Hz, 2H, Ar). ^13^C-NMR δ 26.18, 27.35, 27.41, 29.28, 29.43, 42.30, 55.72, 57.46, 62.38, 68.22, 112.94, 114.65, 115.36, 119.18, 119.77, 120.96, 121.11, 127.16, 127.56, 128.34, 128.49, 129.37, 129.64, 129.72, 130.39, 138.96, 140.11, 144.96, 160.13, 161.44, 190.76. MS (ES) *m*/*z*: 472 [M + H]^+^. C_31_H_37_NO_3_.

*3-(4-((7-(Benzyl(methyl)amino)heptyl)oxy)phenyl)-1-(4-(2-(diethylamino)ethoxy)phenyl)-prop-2-en-1-one* (**3**). Using the previous procedure and starting from **30** and **21**, **3** (yield 70%) was obtained as a solid, mp 40–41 °C (ligroin). ^1^H-NMR δ 1.09 (t, *J* = 7.2 Hz, 6H, 2CH_3_), 1.45–1.55 (m, 4H, 2CH_2_), 1.61–1.82 (m, 2H, CH_2_), 2.19 (s, 3H, NCH_3_), 2.37 (t, *J* = 7.2 Hz, 2H, NCH_2_), 2.63–2.69 (m, 4H, 2CH_2_), 2.91 (t, *J* = 6.4 Hz, 2H, NCH_2_), 3.48 (s, 2H, CH_2_), 4.00 (t, *J* = 6.8 Hz, 2H, OCH_2_), 4.13 (t, *J* = 5.6 Hz, 2H, OCH_2_), 6.92 (d, *J* = 8.8 Hz, 2H, Ar), 6.98 (d, *J* = 9.2 Hz, 2H, Ar), 7.24–7.45 (m, 4H, Ar), 6.92 (d, *J* = 8.8 Hz, 2H, Ar), 7.59 (d, *J* = 8.8 Hz, 2H, Ar), 7.78 (d, *J* = 15.6 Hz, 1H), 8.02 (d, *J* = 8.8 Hz, 2H, Ar). ^13^C-NMR δ 12.37, 26.07, 27.37, 29.20, 29.37, 42.31, 46.51, 46.69, 52.31, 57.51, 62.39, 68.05, 68.24, 113.69, 114.65, 114.84, 115.01, 119.49, 119.75, 120.92, 126.99, 127.49, 127.77, 128.27, 129.18, 129.59, 130.35, 139.19, 140.01, 144.90, 159.40, 161.42, 190.43. MS (ES) *m*/*z*: 557 [M + H]^+^. C_36_H_48_N_2_O_3_.

*3-(4-((7-(Benzyl(methyl)amino)heptyl)oxy)phenyl)-1-(4-(3-(diethylamino)propoxy)phenyl)-prop-2-en-1-one* (**4**). Using the previous procedure and starting from **31** and **21**, **4** (yield 20%) was obtained as an oil. ^1^H-NMR δ 1.01 (t, *J* = 7.2 Hz, 6H, 2CH_3_), 1.45–1.58 (m, 4H, 2CH_2_), 1.61–1.81 (m, 2H, CH_2_), 1.83–2.01 (m, 2H, CH_2_), 2.18 (s, 3H, NCH_3_), 2.36 (t, *J* = 7.2 Hz, 2H, NCH_2_), 2.41–2.65 (m, 6H, 2CH_2_), 3.45 (s, 2H, CH_2_), 3.99 (t, *J* = 6.8 Hz, 2H, OCH_2_), 4.10 (t, *J* = 5.6 Hz, 2H, OCH_2_), 6.92–7.01 (m, 4H, Ar), 7.19–7.42 (m, 6H, Ar), 7.59 (d, *J* = 8.8 Hz, 2H, Ar), 7.78 (d, *J* = 15.6 Hz, 1H), 8.02 (d, *J* = 8.8 Hz, 2H, Ar). ^13^C-NMR δ 11.49, 26.38, 27.51, 28.47, 29.28, 29.45, 42.41, 46.56, 46.82, 52.33, 57.60, 62.96, 68.22, 68.63, 113.74, 114.55, 114.99, 115.36, 119.14, 119.85, 120.92, 126.98, 127.52, 127.78, 128.29, 129.18, 129.61, 130.45, 139.29, 140.22, 144.96, 159.40, 161.02, 190.55. MS (ES) *m*/*z*: 571 [M + H]^+^. HRMS Esi + [M + 1]: calcd for C_37_H_51_N_2_O_3_, 571.3900. Found: 571.3902.

*3-(4-((7-(Benzyl(methyl)amino)heptyl)oxy)phenyl)-1-(4-(4-(diethylamino)butoxy)phenyl)prop-2-en-1-one* (**5**). Using the previous procedure and starting from **32** and **21**, **5** (yield 25%) was obtained as an oil. ^1^H-NMR δ 1.02 (t, *J* = 7.2 Hz, 6H, 2CH_3_), 1.351–1.82 (m, 14H, 7CH_2_), 2.18 (s, 3H, NCH_3_), 2.37 (t, *J* = 7.2 Hz, 2H, NCH_2_), 2.42–2.64 (m, 6H, 2CH_2_), 3.45 (s, 2H, CH_2_), 3.99 (t, *J* = 6.8 Hz, 2H, OCH_2_), 4.10 (t, *J* = 5.6 Hz, 2H, OCH_2_), 6.84–7.03 (m, 4H, Ar), 7.21–7.42 (m, 6H, Ar), 7.60 (d, *J* = 8.8 Hz, 2H, Ar), 7.78 (d, *J* = 15.6 Hz, 1H), 8.01 (d, *J* = 8.8 Hz, 2H, Ar). ^13^C-NMR δ 11.34, 23.27, 26.05, 27.37, 27.48, 29.20, 29.45, 42.37, 46.58, 46.71, 52.36, 57.54, 62.42, 68.25, 68.44, 113.66, 114.65, 114.92, 115.03, 119.48, 119.76, 120.92, 126.99, 127.47, 127.79, 128.27, 129.19, 129.61, 130.37, 139.03, 140.34, 144.92, 159.43, 161.42, 190.55. MS (ES) *m*/*z*: 585 [M + H]^+^. C_38_H_52_N_2_O_3_.

*3-(4-((7-(Benzyl(methyl)amino)heptyl)oxy)phenyl)-1-(3-methoxyphenyl)prop-2-en-1-one* (**12**). Using the previous procedure and starting from 3-methoxyacetophenone and **21**, 1**2** (yield 68%) was obtained as an oil. ^1^H-NMR δ 1.24–1.58 (m, 8H), 1.69–1.82 (m, 2H, CH_2_), 2.19 (s, 3H, NCH_3_), 2.37 (t, *J* = 7.2 Hz, 2H, NCH_2_), 3.44 (s, 2H, CH_2_), 3.88 (s, 3H, OCH_2_), 4.00 (t, *J* = 6.8 Hz, 2H, OCH_2_), 6.88–7.80 (m, 15H, Ar). ^13^C-NMR δ 26.11, 27.30, 27.45, 29.25, 29.40, 42.23, 55.63, 57.44, 62.30, 68.28, 112.94, 114.68, 115.06, 119.19, 119.79, 120.96, 121.09, 127.11, 127.53, 128.34, 128.48, 129.30, 129.64, 129.70, 130.39, 138.96, 140.11, 144.98, 160.01, 161.47, 190.46. MS (ES) *m*/*z*: 472 [M + H]^+^. C_31_H_37_NO_3_.

*3-(4-((7-(Benzyl(methyl)amino)heptyl)oxy)phenyl)-1-(3-(2-(diethylamino)ethoxy)phenyl)-prop-2-en-1-one* (**13**). Using the previous procedure and starting from **33** and **21**, 1**3** (yield 20%) was obtained as an oil. ^1^H-NMR δ 1.10 (t, *J* = 7.2 Hz, 6H, 2CH_3_), 1.20–1.90 (m, 10H, 5CH_2_), 2.20 (s, 3H, NCH_3_), 2.38 (t, *J* = 7.2 Hz, 2H, NCH_2_), 2.60–2.73 (m, 4H, 2CH_2_), 2.90 (t, *J* = 6.4 Hz, 2H, NCH_2_), 3.50 (s, 2H, CH_2_), 4.01 (t, *J* = 6.8 Hz, 2H, OCH_2_), 4.10 (t, *J* = 5.4 Hz, 2H, OCH_2_), 6.98 (d, *J* = 8.8 Hz, 2H, Ar), 7.15–7.82 (m, 13H, Ar). ^13^C-NMR δ 12.37, 26.07, 27.37, 29.20, 29.37, 42.31, 46.51, 46.69, 52.31, 57.51, 62.39, 68.05, 68.24, 113.69, 114.65, 114.84, 115.01, 119.49, 119.75, 120.92, 126.99, 127.49, 127.77, 128.27, 129.18, 129.59, 130.35, 139.19, 140.01, 144.90, 159.40, 161.42, 190.43. MS (ES) *m*/*z*: 557 [M + H]^+^. HRMS Esi + [M + 1]: calcd for C_36_H_49_N_2_O_3_, 557.3743. Found: 557.3741.

*3-(4-((7-(Benzyl(methyl)amino)heptyl)oxy)phenyl)-1-(3-(3-(diethylamino)propoxy)phenyl)-prop-2-en-1-one* (**14**). Using the previous procedure and starting from **34** and **21**, 1**4** (yield 45%) was obtained as an oil. ^1^H-NMR δ 1.01 (t, *J* = 7.2 Hz, 6H, 2CH_3_), 1.20–1.81 (m, 10H, 5CH_2_), 1.82–2.01 (m, 2H, CH_2_), 2.18 (s, 3H, NCH_3_), 2.36 (t, *J* = 7.2 Hz, 2H, NCH_2_), 2.41–2.66 (m, 6H, 3CH_2_), 3.45 (s, 2H, CH_2_), 3.95 (t, *J* = 6.8 Hz, 2H, OCH_2_), 4.05 (t, *J* = 5.8 Hz, 2H, OCH_2_), 6.79–7.80 (m, 15H, Ar). ^13^C-NMR δ 11.47, 26.35, 27.37, 28.01, 29.24, 29.37, 42.38, 46.51, 46.76, 52.29, 57.52, 62.93, 68.15, 68.24, 113.55, 114.47, 114.91, 115.19, 119.04, 119.75, 120.92, 126.99, 127.49, 127.77, 128.27, 129.18, 129.59, 130.38, 139.19, 140.14, 144.90, 159.40, 160.98, 190.36. MS (ES) *m*/*z*: 571 [M + H]^+^. C_37_H_50_N_2_O_3_.

*3-(4-((7-(Benzyl(methyl)amino)heptyl)oxy)phenyl)-1-(4-(4-(diethylamino)butoxy)phenyl)prop-2-en-1-one* (**15**). Using the previous procedure and starting from **35** and **21**, 1**5** (yield 55%) was obtained as an oil. ^1^H-NMR δ 1.02 (t, *J* = 7.2 Hz, 6H, 2CH_3_), 1.22–1.60 (m, 10H, 5CH_2_), 1.61–1.90 (m, 4H, 2CH_2_), 2.19 (s, 3H, NCH_3_), 2.38 (t, *J* = 7.2 Hz, 2H, NCH_2_), 2.42–2.59 (m, 6H, 3CH_2_), 3.42 (s, 2H, CH_2_), 3.99–4.13 (m, 4H, 2OCH_2_), 6.85 (d, *J* = 8.8 Hz, 2H, Ar), 7.10–7.82 (m, 13H, Ar). ^13^C-NMR δ 11.35, 23.21, 26.07, 27.37, 27.41, 29.20, 29.37, 42.31, 46.51, 46.69, 52.31, 57.51, 62.39, 68.05, 68.24, 113.69, 114.65, 114.84, 115.01, 119.49, 119.75, 120.92, 126.99, 127.49, 127.77, 128.27, 129.18, 129.59, 130.35, 139.19, 140.01, 144.90, 159.40, 161.42, 190.43. MS (ES) *m*/*z*: 585 [M + H]^+^. C_38_H_52_N_2_O_3_.

*3-(4-((7-(Benzyl(methyl)amino)heptyl)oxy)phenyl)-1-(4-(2-chloroethoxy)phenyl)prop-2-en-1-one* (**36**). Using the previous procedure and starting from **24** and **21**, **36** (yield 77%) was obtained as yellow oil. ^1^H-NMR δ 1.22–1.80 (m, 10H), 2.18 (s, 3H), 2.35 (t, *J* = 7.2 Hz, 2H), 3.45 (s, 2H), 3.80 (t, 2H), 3.99 (t, 2H), 4.30 (t, 2H), 6.85–7.80 (m, 13H, Ar), 8.02 (d, 2H).

General method for the synthesis of final compounds **6**–**11**: A stirred solution of **36** (0.5 mmol) and the selected amine (1 mmol) in toluene (50 mL) was refluxed for 24 h. The mixture was washed with water (3 × 25 mL) and the organic layer was dried. The solvent was removed under reduced pressure and the residue was purified by flash chromatography on silica gel (toluene/acetone 4:1, then only MeOH).

*3-(4-((7-(Benzyl(methyl)amino)heptyl)oxy)phenyl)-1-(4-(2-morpholinoethoxy)phenyl)prop-2-en-1-one* (**6**). Using the previous procedure and starting from morpholine, **6** (yield 15%) was obtained as an oil. ^1^H-NMR δ 1.25–1.90 (m, 10H, 5CH_2_), 2.18 (s, 3H, NCH_3_), 2.35 (t, *J* = 7.2 Hz, 2H, NCH_2_), 2.43–2.61 (m, 4H, 2NCH_2_), 2.83 (t, 2H, NCH_2_), 3.42 (s, 2H, CH_2_), 3.70–3.80 (m, 4H, 2OCH_2_), 3.99 (t, *J* = 6.8 Hz, 2H, OCH_2_), 4.15 (t, *J* = 6.8 Hz, 2H, OCH_2_), 6.80–8.03 (m, 15H, Ar). ^13^C-NMR δ 26.42, 27.33, 27.45, 29.25, 29.40, 42.32, 56.21, 57.54, 57.56, 57.86, 62.38, 66.71, 66.73, 67.98, 68.26, 114.47, 115.21, 114.84, 119.49, 119.55, 127.16, 127.74, 127.77, 128.37, 129.28, 130.24, 130.82, 138.69, 140.34, 144.23, 159.62, 162.65, 189.97. MS (ES) *m*/*z*: 571 [M + H]^+^. HRMS Esi + [M + 1]: calcd for C_36_H_47_N_2_O_4_, 571.3536. Found: 571.3535.

*3-(4-((7-(Benzyl(methyl)amino)heptyl)oxy)phenyl)-1-(4-(2-(piperidin-1-yl)ethoxy)phenyl)prop-2-en-1-one* (**7**). Using the previous procedure and starting from piperidine, **7** (yield 95%) was obtained as a solid, mp 58–59 °C (ligroin). ^1^H-NMR δ 1.20–1.90 (m, 16H, 8CH_2_), 2.18 (s, 3H, NCH_3_), 2.33 (t, *J* = 7.2 Hz, 2H, NCH_2_), 2.41–2.59 (m, 4H, 2NCH_2_), 2.79 (t, *J* = 6.8 Hz, 2H, NCH_2_), 3.43 (s, 2H, CH_2_), 3.99 (t, *J* = 6.8 Hz, 2H, OCH_2_), 4.18 (t, *J* = 6.8 Hz, 2H, OCH_2_), 6.82–7.90 (m, 13H, Ar), 8.01 (d, *J* = 8.8 Hz, 2H, Ar). ^13^C-NMR δ 24.26, 26.02, 26.12, 27.01, 27.39, 27.45, 29.26, 29.41, 42.34, 55.21, 57.54, 57.86, 62.41, 66.35, 68.26, 114.49, 115.01, 114.84, 119.49, 119.54, 127.06, 127.74, 127.77, 128.33, 129.24, 130.24, 130.80, 138.69, 140.11, 144.03, 159.61, 162.65, 188.91. MS (ES) *m*/*z*: 569 [M + H]^+^. HRMS Esi + [M + 1]: calcd for C_37_H_49_N_2_O_3_, 569.3743. Found: 569.3744.

*3-(4-((7-(Benzyl(methyl)amino)heptyl)oxy)phenyl)-1-(4-(2-(3,4-dihydroisoquinolin-2(1H)-yl)ethoxy)phenyl)prop-2-en-1-one* (**8**). Using the previous procedure and starting from 1,2,3,4-tetrahydroisoquinoline, **8** (yield 30%) was obtained as an oil. ^1^H-NMR δ 1.22–1.80 (m, 10H, 5CH_2_), 2.18 (s, 3H, NCH_3_), 2.31 (t, *J* = 7.2 Hz, 2H, NCH_2_), 2.80–3.02 (m, 6H, 3NCH_2_), 3.45 (s, 2H, CH_2_), 3.79 (s, 2H, CH_2_), 3.99 (t, *J* = 6.8 Hz, 2H, OCH_2_), 4.26 (t, *J* = 6.6 Hz, 2H, OCH_2_), 6.86–7.85 (m, 17H, Ar), 8.03 (d, *J* = 8.8 Hz, 2H, Ar). ^13^C-NMR δ 25.91, 26.74, 27.22, 29.06, 29.17, 41.74, 51.39, 56.38, 57.01, 61.83, 67.04, 68.07, 114.18, 114.35, 119.29, 125.68, 126.25, 126.57, 126.95, 127.04, 127.27, 128.29, 129.37, 130.11, 130.68, 130.73, 131.81, 132.17, 138.27, 145.19, 158.42, 162.20, 192.42. MS (ES) *m*/*z*: 617 [M + H]^+^. C_41_H_48_N_2_O_3_.

*1-(1-(2-(4-(3-(4-((7-(Benzyl(methyl)amino)heptyl)oxy)phenyl)acryloyl)phenoxy)ethyl)-piperidin-4-yl)-1,3-dihydro-2H-benzo[d]imidazol-2-one* (**9**). Using the previous procedure and starting from 1-(piperidin-4-yl)-1,3-dihydro-2H-benzo[d]imidazol-2-one, **9** (yield 70%) was obtained as an oil. ^1^H-NMR δ 1.20–1.60 (m, 8H, 4CH_2_), 1.60–1.95 (m, 2H, CH_2_), 2.18 (s, 3H, NCH_3_), 2.24–2.60 (m, 4H, 2NCH_2_), 2.90 (t, *J* = 6.8 Hz, 2H, NCH_2_), 3.10–3.20 (m, 2H, NCH_2_), 3.43 (s, 2H, CH_2_), 3.99 (t, *J* = 6.8 Hz, 2H, OCH_2_), 4.20 (t, *J* = 6.8 Hz, 2H, OCH_2_), 4.25–4.38 (m, 1H), 6.82–7.82 (m, 17H, Ar), 8.02 (d, *J* = 8.8 Hz, 2H, Ar), 9.10 (br, 1H, NH). ^13^C-NMR δ 26.02, 26.18, 27.11, 27.38, 27.46, 29.26, 29.41, 42.34, 55.21, 57.54, 57.86, 59.01, 62.41, 66.34, 68.26, 111.76, 114.49, 115.13, 114.84, 119.49, 119.54, 124.51, 124.56, 126.81, 127.06, 127.74, 127.77, 128.33, 128.89, 129.24, 130.24, 130.80, 138.69, 140.11, 144.03, 152.62, 159.61, 162.65, 188.91. MS (ES) m/z: 701 [M + H]^+^. C_44_H_52_N_4_O_4_.

*3-(4-((7-(Benzyl(methyl)amino)heptyl)oxy)phenyl)-1-(4-(2-(4-phenylpiperidin-1-yl)ethoxy)-phenyl)prop-2-en-1-one* (**10**). Using the previous procedure and starting from phenylpiperidine, **10** (yield 65%) was obtained as a solid, mp 81–82 °C (ligroin). ^1^H-NMR δ 1.20–1.61 (m, 10H, 5CH_2_), 1.62–1.97 (m, 4H, 2CH_2_), 2.18 (s, 3H, NCH_3_), 2.20–2.61 (m, 5H), 2.90 (t, *J* = 6.8 Hz, 2H, NCH_2_), 3.05–3.20 (m, 2H), 3.46 (s, 2H, CH_2_), 3.99 (t, *J* = 6.8 Hz, 2H, OCH_2_), 4.20 (t, *J* = 6.8 Hz, 2H, OCH_2_), 6.86–7.82 (m, 18H, Ar). 8.02 (d, *J* = 8.8 Hz, 2H, Ar). ^13^C-NMR δ 26.18, 27.11, 27.46, 29.26, 29.41, 30.21, 30.26, 42.07, 42.44, 55.21, 57.54, 57.86, 62.41, 66.34, 68.26, 111.76, 114.49, 115.13, 114.84, 119.49, 119.54, 124.51, 124.56, 126.81, 127.06, 127.74, 127.77, 128.36, 128.89, 129.24, 130.25, 130.80, 138.69, 140.11, 144.03, 159.66, 162.65, 189.75. MS (ES) *m*/*z*: 645 [M + H]^+^. C_43_H_52_N_2_O_3_.

*3-(4-((7-(Benzyl(methyl)amino)heptyl)oxy)phenyl)-1-(4-(2-(4-(4-chlorophenyl)-4-hydroxypiperidin-1-yl)ethoxy)phenyl)prop-2-en-1-one* (**11**). Using the previous procedure and starting from 4-(4-chlorophenyl)piperidin-4-ol, **11** (yield 15%) was obtained as an oil. ^1^H-NMR δ 1.20–1.61 (m, 10H, 5CH_2_), 1.62–1.90 (m, 4H, 2CH_2_), 2.05–2.15 (m, 2H), 2.18 (s, 3H, NCH_3_), 2.30 (t, *J* = 6.8 Hz, 2H, NCH_2_), 2.54–2.61 (m, 2H), 2.91 (t, 2H, NCH_2_), 3.41 (s, 2H, CH_2_), 3.99 (t, 2H, OCH_2_), 4.20 (t, 2H, OCH_2_), 6.86–7.79 (m, 17H, Ar), 8.02 (d, *J* = 8.8 Hz, 2H, Ar). ^13^C-NMR δ 26.78, 27.12, 27.48, 29.26, 29.44, 30.24, 30.30, 42.44, 55.28, 57.53, 57.87, 62.41, 66.37, 68.26, 69.88, 111.66, 114.51, 115.14, 114.84, 119.49, 119.54, 124.51, 124.56, 127.06, 127.74, 127.77, 128.36, 128.89, 129.24, 130.25, 130.80, 131.63, 138.69, 140.11, 144.03, 159.66, 162.65, 189.75. MS (ES) m/z: 696 [M + H]^+^. C_43_H_51_ClN_2_O_4_.

General method for the synthesis of final compounds **16**–**18**: A solution of **2** or **3** or **4** (0.1 mmol) in THF (50 mL) was hydrogenated at room temperature and pressure over Pd/CaCO_3_. The solution was filtered from catalyst and evaporated to dryness. The residue was purified by flash chromatography on silica gel (only MeOH).

*3-(4-((7-(Benzyl(methyl)amino)heptyl)oxy)phenyl)-1-(4-(2-(diethylamino)ethoxy)phenyl)-propan-1-one* (**16**). Using the previous procedure and starting from **2**, **16** (yield 95%) was obtained as an oil. ^1^H-NMR δ 1.05 (t, *J* = 7.2 Hz, 6H, 2CH_3_), 1.20–1.61 (m, 8H, 4CH_2_), 1.63–1.82 (m, 2H, CH_2_), 2.19 (s, 3H, NCH_3_), 2.38 (t, *J* = 7.2 Hz, 2H, NCH_2_), 2.58–2.69 (m, 4H, 2CH_2_), 2.85–3.05 (m, 4H, 2CH_2_), 3.18 (t, *J* = 8.6 Hz, 2H, NCH_2_), 3.46 (s, 2H, CH_2_), 3.97 (t, *J* = 8.6 Hz, 2H, OCH_2_), 4.15 (t, *J* = 8.6 Hz, 2H, OCH_2_), 6.63 (s, 1H), 6.80–7.35 (m, 10H, Ar), 7.98 (d, *J* = 8.8 Hz, 2H, Ar). ^13^C-NMR δ 12.37, 26.07, 27.37, 29.20, 29.37, 35.08, 40.65, 42.31, 46.51, 46.69, 52.31, 57.51, 62.39, 68.05, 68.24, 113.69, 114.65, 114.84, 115.01, 119.49, 119.75, 126.99, 127.49, 127.77, 128.27, 129.18, 129.59, 130.35, 139.19, 140.01, 159.40, 161.42, 197.83. MS (ES) *m*/*z*: 560 [M + H]^+^. C_36_H_50_N_2_O_3_.

*3-(4-((7-(Benzyl(methyl)amino)heptyl)oxy)phenyl)-1-(4-(3-(diethylamino)propoxy)phenyl)-propan-1-one* (**17**). Using the previous procedure and starting from **3**, **17** (yield 95%) was obtained as an oil. ^1^H-NMR δ 1.01 (t, *J* = 7.2 Hz, 6H, 2CH_3_), 1.20–1.61 (m, 8H, 4CH_2_), 1.63–1.81 (m, 2H, CH_2_), 1.85–2.02 (m, 2H, CH_2_), 2.18 (s, 3H, NCH_3_), 2.35 (t, *J* = 7.2 Hz, 2H, NCH_2_), 2.41–2.64 (m, 6H), 2.99 (t, *J* = 8.6 Hz, 2H), 3.19 (t, *J* = 8.6 Hz, 2H), 3.45 (s, 2H, CH_2_), 3.90–4.08 (m, 4H, 2OCH_2_), 6.78–7.83 (m, 10H, Ar), 7.95 (d, *J* = 12.6 Hz, 2H), 8.02 (d, *J* = 8.8 Hz, 1H, Ar). ^13^C-NMR δ 11.37, 26.05, 27.37, 27.84, 29.24, 29.37, 35.15, 40.77, 42.38, 46.51, 46.76, 52.29, 57.52, 62.41, 68.15, 68.26, 113.55, 114.47, 114.91, 115.19, 119.04, 119.75, 126.99, 127.49, 127.77, 128.27, 129.18, 129.59, 130.41, 139.24, 140.14, 159.40, 160.98, 197.86. MS (ES) *m*/*z*: 573 [M + H]^+^. C_37_H_52_N_2_O_3_.

*3-(4-((7-(Benzyl(methyl)amino)heptyl)oxy)phenyl)-1-(4-(4-(diethylamino)butoxy)phenyl)-propan-1-one* (**18**). Using the previous procedure and starting from **4**, **18** (yield 90%) was obtained as an oil. ^1^H-NMR δ 1.05 (t, *J* = 7.2 Hz, 6H, 2CH_3_), 1.20–1.85 (m, 14H, 7CH_2_), 2.19 (s, 3H, NCH_3_), 2.38 (t, 2H, NCH_2_), 2.58–2.69 (m, 6H), 2.95–3.05 (m, 2H), 3.18–3.24 (m, 2H), 3.46 (s, 2H, CH_2_), 4.00 (t, *J* = 8.6 Hz, 2H, OCH_2_), 4.11 (t, *J* = 8.6 Hz, 2H, OCH_2_), 6.80–7.21 (m, 11H, Ar), 7.98 (d, *J* = 8.8 Hz, 2H, Ar). ^13^C-NMR δ 11.69, 23.21, 26.17, 27.32, 29.46, 29.65, 35.04, 40.54, 42.39, 46.89, 46.69, 52.57, 57.63, 62.47, 68.12, 68.24, 113.72, 114.31, 114.67, 115.22, 119.39, 119.65, 126.98, 127.51, 127.84, 128.30, 129.20, 129.42, 130.43, 139.29, 140.11, 156.20, 160.42, 198.22. MS (ES) *m*/*z*: 587 [M + H]^+^. C_38_H_54_N_2_O_3_.

*4-(7-Bromoheptyloxy)benzonitrile* (**19**). A stirred mixture of 4-hydroxybenzonitrile (2 g, 0.017 mol), 1,7-dibromoheptane (4.3 mL, 0.025 mol) and K_2_CO_3_ (4 g) was refluxed in acetone (150 mL) for 20 h. The suspension was filtered while hot, and the solvent was removed under reduced pressure. After adding petroleum ether, the residue was kept in the freezer overnight and the white solid that formed was filtered off, affording **19** (1.41 g, 70%). mp 47–49 °C (ligroin). ^1^H-NMR δ 1.46–1.98 (m, 10H), 3.41 (t, *J* = 6.8 Hz, 2H), 3.99 (t, *J* = 6.8 Hz, 2H), 6.5 (d, *J* = 8.8 Hz, 2H, Ar), 7.61 (d, 2H, *J* = 6.8 Hz, Ar).

*4-((7-(Benzyl(methyl)amino)heptyl)oxy)benzonitrile* (**20**). A stirred solution of **19** (1.14 g, 3.85 mmol), *N*-benzyl-*N*-methylamine (1.5 mL, 3.8 mmol) and TEA (0.4 mL, 3.8 mmol) in toluene (100 mL) was refluxed for 20 h. The mixture was washed with water (3 × 25 mL) and the organic layer was dried. The solvent was removed under reduced pressure and the residue was purified by flash chromatography on silica gel (petroleum ether/ethyl acetate 9:1), affording **20** as an oil (0.9 g, 70%). ^1^H-NMR δ 1.30–1.58 (m, 8H), 1.71–1.82 (m, 2H), 2.18 (s, 3H), 2.37 (t, *J* = 7.2 Hz, 2H), 3.45 (s, 2H), 3.97 (t, *J* = 6.8 Hz, 2H), 6.88 (d, *J* = 8.8 Hz, 2H, Ar), 7.19–7.30 (m, 5H, Ar), 7.55 (d, *J* = 8.8 Hz, 2H, Ar).

*4-((7-(Benzyl(methyl)amino)heptyl)oxy)benzaldehyde* (**21**). A mixture of **20** (2.18 g, 6.48 mmol) and Ni/Raney alloy (3.06 g, 13 mmol) in 75% HCOOH (44 mL) was refluxed for 7 h, and then hot filtered. The residue was diluted with 100–150 mL of water, basified by K_2_CO_3_ and extracted with DCM (3 × 25 mL). The combined organic extracts were dried, and concentrated under reduced pressure to afford **21** (1.9 g, 90%) as yellow oil (purified by flash chromatography with toluene/acetone 9:1 as eluent). ^1^H-NMR δ 1.22–1.59 (m, 8H), 1.65–1.83 (m, 2H), 2.18 (s, 3H), 2.38 (t, *J* = 7.2 Hz, 2H), 3.43 (s, 2H), 4.01 (t, *J* = 6.8 Hz, 2H), 6.94–6.99 (m, 2H, Ar), 7.18–7.35 (m, 5H, Ar), 7.79 (d, *J* = 8.8 Hz, 2H, Ar), 9.85 (broad, 1H).

*1-(4-(Bromomethyl)phenyl)ethan-1-one* (**22**). A mixture of 4-methylacetophenone (2 g, 0.015 mol), *N-*bromosuccinimide (NBS, 2.66 g, 0.015 mol) and a catalytic amount of benzoyl peroxide in CCl_4_ was refluxed for 4 h. The mixture was hot filtered and evaporated to dryness to afford **22** (2.9 g, 95%) as brown oil. ^1^H-NMR δ 2.51 (s, 3H), 4.52 (s, 2H), 7.40 (d, *J* = 8.8 Hz, 2H, Ar), 7.91 (d, *J* = 8.8 Hz, 2H, Ar).

*1-(4-((Diethylamino)methyl)phenyl)ethan-1-one* (**23**). A stirred solution of **22** (1.5 g, 0.007 mol) and diethylamine (2.2 mL, 0.021 mol) in toluene (30 mL) was refluxed for 24 h. The mixture was washed with water (3 × 25 mL) and the organic layer was dried. The solvent was removed under reduced pressure and the residue was purified by flash chromatography on silica gel (petroleum ether/ethyl acetate 9:1), affording **23** as an oil used for the next step without further purification. ^1^H-NMR δ 1.01 (t, *J* = 6.8 Hz, 6H), 2.43–2.59 (m, 7H), 3.60 (s, 2H), 7.40 (d, *J* = 8.8 Hz, 2H, Ar), 7.90 (d, *J* = 8.8 Hz, 2H, Ar).

General method for the synthesis of compounds **24**–**29**: A stirred mixture of 4-hydroxyacetophenone (0.022 mol), selected bromochloroalkane (0.044 mol) and K_2_CO_3_ (6 g) was refluxed in acetone (150 mL) for 20 h. The suspension was filtered while hot, and the solvent was removed under reduced pressure. After adding petroleum ether, the residue was kept in the freezer overnight and the white solid that formed was filtered off and purified by flash chromatography on silica gel (petroleum ether/ethyl acetate 9:1).

*1-(4-(2-Chloroethoxy)phenyl)ethan-1-one* (**24**). Using the previous procedure and starting from 1-bromo-2-chloroethane, **24** (yield 50%) was obtained as a white solid, mp 59–60 °C (ligroin). ^1^H-NMR δ 2.58 (s, 3H), 3.85 (t, *J* = 6.8 Hz, 2H), 4.25 (t, 2H), 6.95 (d, *J* = 8.8 Hz, 2H, Ar), 7.95 (d, *J* = 8.8 Hz, 2H, Ar).

*1-(4-(3-Chloropropoxy)phenyl)ethan-1-one* (**25**). Using the previous procedure and starting from 1-chloro-3-bromopropane, **25** (yield 78%) was obtained as a white solid, mp 25–26 °C (ligroin). ^1^H-NMR δ 2.11–2.35 (m, 2H), 2.57 (s, 3H), 3.72 (t, *J* = 6.8 Hz, 2H), 4.18 (t, *J* = 6.8 Hz, 2H), 6.90 (d, *J* = 8.8 Hz, 2H, Ar), 7.95 (d, *J* = 8.8 Hz, 2H, Ar).

*1-(4-(4-Chlorobutoxy)phenyl)ethan-1-one* (**26**). Using the previous procedure and starting from 1-chloro-4-bromobutane, **26** (yield 80%) was obtained as a white solid, mp 39–40 °C (ligroin). ^1^H-NMR δ 1.98–2.05 (m, 4H), 2.58 (s, 3H), 3.62 (t, *J* = 6.8 Hz, 2H), 4.17 (t, *J* = 6.8 Hz, 2H), 6.90 (d, *J* = 8.8 Hz, 2H, Ar), 7.91 (d, *J* = 8.8 Hz, 2H, Ar).

*1-(3-(2-Chloroethoxy)phenyl)ethan-1-one* (**27**). Using the previous procedure and starting from 3-hydroxyacetophenone and 1-bromo-2-chloroethane, **27** (yield 45%) was obtained as an oil. ^1^H-NMR δ 2.59 (s, 3H), 3.81 (t, *J* = 6.8 Hz, 2H), 4.27 (t, *J* = 6.8 Hz, 2H), 7.05–7.60 (m, 4H, Ar).

*1-(3-(3-Chloropropoxy)phenyl)ethan-1-one* (**28**). Using the previous procedure and starting from 3-hydroxyacetophenone and 1-chloro-3-bromopropane, **28** (yield 70%) was obtained as yellow oil. ^1^H-NMR δ 2.12–2.36 (m, 2H), 2.57 (s, 3H), 3.71 (t, *J* = 6.8 Hz, 2H), 4.17 (t, *J* = 6.8 Hz, 2H), 7.05–7.58 (m, 4H, Ar).

*1-(3-(4-Chlorobutoxy)phenyl)ethan-1-one* (**29**). Using the previous procedure and starting from 3-hydroxyacetophenone and 1-chloro-4-bromobutane, **29** (yield 50%) was obtained as a white solid, mp 25–26 °C (ligroin). ^1^H-NMR δ 1.98–2.05 (m, 4H), 2.58 (s, 3H), 3.60 (t, *J* = 6.8 Hz, 2H), 4.06 (t, *J* = 6.8 Hz, 2H), 7.06–7.56 (m, 4H, Ar).

General method for the synthesis of compounds **30**–**35**. A stirred solution of selected chloroderivative (6.5 mmol) and diethylamine (13 mmol) in toluene (100 mL) was refluxed for 24 h. The mixture was washed with water (3 × 25 mL) and the organic layer was dried. The solvent was removed under reduced pressure and the residue was purified by flash chromatography on silica gel (toluene/acetone 4:1).

*1-(4-(2-(Diethylamino)ethoxy)phenyl)ethan-1-one* (**30**). Using the previous procedure and starting from **24**, **30** (yield 40%) was obtained as an oil. ^1^H-NMR δ 1.03 (t, *J* = 7.2 Hz, 6H), 2.48–2.70 (m, 7H), 2.89 (t, *J* = 6.8 Hz, 2H), 4.05 (t, *J* = 6.8 Hz, 2H), 6.85 (d, *J* = 8.8 Hz, 2H, Ar), 7.90 (d, *J* = 8.8 Hz, 2H, Ar).

*1-(4-(3-(Diethylamino)propoxy)phenyl)ethan-1-one* (**31**). Using the previous procedure and starting from **25**, **31** (yield 70%) was obtained as an oil. ^1^H-NMR δ 1.03 (t, *J* = 7.2 Hz, 6H), 1.85–2.02 (m, 2H), 2.45–2.64 (m, 9H), 4.09 (t, *J* = 6.8 Hz, 2H), 6.90 (d, *J* = 8.8 Hz, 2H, Ar), 7.90 (d, *J* = 8.8 Hz, 2H, Ar).

*1-(4-(4-(Diethylamino)butoxy)phenyl)ethan-1-one* (**32**). Using the previous procedure and starting from **26**, **32** (yield 90%) was obtained as an oil. ^1^H-NMR δ 1.03 (t, *J* = 7.2 Hz, 6H), 1.61–1.89 (m, 4H), 2.43–2.61 (m, 9H), 4.02 (t, *J* = 6.8 Hz, 2H), 6.91 (d, *J* = 8.8 Hz, 2H, Ar), 7.90 (d, *J* = 8.8 Hz, 2H, Ar).

*1-(3-(2-(Diethylamino)ethoxy)phenyl)ethan-1-one* (**33**). Using the previous procedure and starting from **27**, **33** (yield 40%) was obtained as yellow oil. ^1^H-NMR δ 1.06 (t, *J* = 7.2 Hz, 6H), 2.59–2.72 (m, 7H), 2.95 (t, *J* = 6.8 Hz, 2H), 4.08 (t, *J* = 6.8 Hz, 2H), 7.08–7.58 (m, 4H, Ar).

*1-(3-(3-(Diethylamino)propoxy)phenyl)ethan-1-one* (**34**). Using the previous procedure and starting from **28**, **34** (yield 90%) was obtained as an oil. ^1^H-NMR δ 1.02 (t, *J* = 7.2 Hz, 6H), 1.88–1.99 (m, 2H), 2.58–2.64 (m, 9H), 4.01 (t, *J* = 6.8 Hz, 2H), 7.06–7.55 (m, 4H, Ar).

*1-(3-(4-(Diethylamino)butoxy)phenyl)ethan-1-one* (**35**). Using the previous procedure and starting from **29**, **35** (yield 50%) was obtained as an oil. ^1^H-NMR δ 1.01 (t, *J* = 7.2 Hz, 6H), 1.52–1.90 (m, 4H), 2.40–2.60 (m, 9H), 4.01 (t, *J* = 6.8 Hz, 2H), 7.07–7.57 (m, 4H, Ar).

### 3.2. Inhibition of Human Cholinesterases

The capacity of **1**–**18** to inhibit human cholinesterase activity was assessed using the Ellman’s assay [16]. The assays were performed on a Jasco V-530 double beam spectrophotometer connected to a HAAKE DC30 thermostating system (Thermo Haake, Karlsruhe, Germany). Stock solutions of the tested compound (2 mM) were prepared in methanol and diluted in methanol. The assay solution consisted of a 0.1 M phosphate buffer, pH 8.0, with the addition of 340 μM 5,5′-dithiobis(2-nitrobenzoic acid), 0.02 unit/mL human recombinant AChE or BuChE from human serum (Sigma-Aldrich, Milan, Italy), and 550 μM substrate, i.e., acetylthiocholine iodide or butyrylthiocholine iodide, respectively (Sigma-Aldrich). Inhibitors were added to the assay solution at increasing concentrations and preincubated at 37 °C with the enzyme for 20 min before the addition of substrate. The rate of absorbance increase at 412 nm was followed for 3 min. In parallel, blanks containing all components except the enzyme were prepared to account for the non-enzymatic hydrolysis of the substrate. The reaction rates were compared and the percent inhibition due to the presence of the tested compound was calculated. Each concentration was analyzed at least in duplicate. Inhibition plots for each compound were obtained by plotting the % inhibition versus the logarithm of inhibitor concentration in the assay solution. The linear regression parameters were determined for each curve and the IC_50_ was extrapolated. For each tested compound two independent assessments of the IC_50_ value were carried out.

### 3.3. Cell Culture

Human neuronal (SH-SY5Y) cells were routinely grown in Dulbecco’s modified Eagle’s Medium supplemented with 10% fetal bovine serum, 2 mM l-glutamine, 50 U/mL penicillin and 50 µg/mL streptomycin at 37 °C in a humidified incubator with 5% CO_2_.

### 3.4. Determination of Neurotoxicity

To establish the range of concentrations not associated with neurotoxicity, SH-SY5Y cells were seeded in a 96-well plate at 2 × 10^4^ cells/well, incubated for 24 h and subsequently treated with different concentrations of compounds **2**, **4** and **17** (2.5–80 µM) for 24 h at 37 °C in 5% CO_2_. Neuronal cells viability, in terms of mitochondrial metabolic function, was evaluated by the reduction of 3-(4,5-dimethyl-2-thiazolyl)-2,5-diphenyl-2H-tetrazolium bromide (MTT) to its insoluble formazan, as previously described [26]. Briefly, the treatment medium was replaced with MTT in Hank’s Balanced Salt Solution (HBSS) (0.5 mg/mL) for 2 h at 37 °C in 5% CO_2_. After washing with HBSS, formazan crystals were dissolved in isopropanol. The amount of formazan was measured (570 nm, reference filter 690 nm) using a multilabel plate reader (VICTOR™ X3, PerkinElmer, Waltham, MA, USA). The quantity of formazan was directly proportional to the number of viable cells.

### 3.5. Determination of Antioxidant Activity

The intracellular antioxidant activity of the studied compounds was evaluated in SH-SY5Y cells as previously described [27]. Briefly, SH-SY5Y cells were seeded in a 96-well plate at 2 × 10^4^ cells/well and incubated for 24 h at 37 °C in 5% CO_2_. Subsequently, SH-SY5Y cells were incubated for 24 h with compounds **2**, **4** and **17** (1.25 µM). At the end of incubation, the treatment medium was removed and 100 µL of a fluorescent probe, 2′-7′ dichlorodihydrofluorescein diacetate (H_2_DCF-DA) (10 µg/mL), was added to each well. After 30 min of incubation at room temperature, H_2_DCF-DA solution was replaced with a solution of *tert*-butyl hydroperoxide (*t*-BuOOH) (100 µM) for 30 min. The reactive oxygen species (ROS) formation was measured (excitation at 485 nm and emission at 535 nm) using a multilabel plate reader (VICTOR™ X3, PerkinElmer). The antioxidant activity in terms of inhibition percentage in ROS formation induced by *t*-BuOOH, is calculated using the following formula:

(1) $$\%\;\mathrm{of}\;\mathrm{inhibition}=100-\left(\frac{Ptc\;\times \;100}{Pt}\right)$$

where *Ptc* = % of increase in ROS formation induced by *t*-BuOOH in the presence of the studied compounds; *Pt* = % of increase in ROS formation induced by *t*-BuOOH.

### 3.6. Determination of Glutathione Levels

The GSH levels were evaluated in SH-SY5Y cells as previously described [28]. SH-SY5Y cells were seeded in a black 96-well plate at 2 × 10^4^ cells/well and incubated for 24 h at 37 °C in 5% CO_2_. Subsequently, SH-SY5Y cells were incubated for 24 h with compounds **2**, **4** and **17** [1.25 µM]. At the end of incubation, the treatment medium was removed, and 100 µL of a fluorescent probe, monochlorobimane (MCB), was added to each well. After 30 min of incubation at 37 °C in 5% CO_2_, the GSH levels were measured (excitation at 355 nm and emission at 460 nm) using a multilabel plate reader (VICTOR™ X3, PerkinElmer). Data are expressed as concentration of GSH (µM) obtained by a GSH standard curve.

### 3.7. Aβ_1–42_ Oligomers Preparation for the Determination of Neuroprotective Activity

Aβ_1–42_ peptide was first dissolved in 1,1,1,3,3,3,-hexafluoroisopropanol to 1 mg/mL, sonicated, incubated at room temperature for 24 h and lyophilized. The resulting unaggregated Aβ_1–42_ peptide film was dissolved with DMSO and stored at −20 °C until use. The Aβ_1–42_ peptide aggregation to oligomeric form was prepared as previously described [29].

### 3.8. Determination of Neuroprotective Activity toward Aβ_1–42_ Oligomers

To evaluate the neuroprotective activity of the studied compounds, SH-SY5Y cells were seeded in a 96-well plate at 3 × 10^4^ cells/well, incubated for 24 h and subsequently treated with Aβ_1–42_ oligomers (OAβ_1–42_) [10 µM] in the presence of compounds **2**, **4** and **17** [1.25 µM] for 4 h. The neuroprotective activity, in terms of increase in intracellular MTT granules, was measured by MTT formazan exocytosis assay, as previously described [30]. Briefly, the treatment medium was replaced with MTT in HBSS (0.5 mg/mL) for 1 h at 37 °C in 5% CO_2_. After the incubation, intracellular MTT granules were completely solubilized in Tween-20 (10% *v*/*v*). The absorbance of Tween-20 soluble MTT was measured at 570 nm (reference filter 690 nm) using a multilabel plate reader (VICTOR™ X3, PerkinElmer). Data are expressed as percentage of neuronal viability versus control.

### 3.9. Docking Studies

Molecular dockings were performed by using the Autodock Vina [31] software 1, The Scripps Research Institute., La Jolla, CA, USA). Diprotonated compounds **4** and **6** were prepared with Discovery Studio, version 2.1, software package (BIOVIA, San Diego, CA, USA), using standard bond lengths and bond angles. With the CHARMm force field [32] and partial atomic charges, the molecular geometries of the compounds were energy-minimized using the adopted-based Newton-Rapson algorithm until the rms gradient was below 0.01 kcal (mol Å)^−1^.

The crystal structure of hAChE complexed with fasciculin-II (PDB: 1B41) was obtained from the Protein Data Bank (PDB). The protein was prepared by removing all water molecules, heteroatoms, any co-crystallized solvent and the ligand. Protein model tool in Discovery Studio, version 2.1, software package was used to assign proper bonds, bond orders, hybridization and charges. CHARMm force field was applied using the receptor-ligand interactions tool in Discovery Studio, version 2.1, software package. AutoDockTools (ADT; version 1.5.4, The Scripps Research Institute, La Jolla, CA, USA) was used to add hydrogens and partial charges for proteins and ligands using Gasteiger charges. The structures of the ligands were then loaded in ADT Tools and flexible torsions were assigned with AutoTors module, and the acyclic dihedral angles were allowed to rotate freely. Some selected side chains into the hAChE macromolecule were also allowed to change their conformations. Using the AutoTors module, the macromolecule side chains chosen to be flexible are: Trp286, Tyr124, Tyr337, Tyr72, Asp74, Thr75, Trp86 and Tyr341. The docking box was displayed using ADT and it is big enough to include the whole protein target (“blind docking”). A grid box of 60 × 60 × 72 with grid point spacing of 1 Ǻ, was positioned at the middle of the protein (x = 116.546; y = 110.33; z = −134.181).

The three-dimensional structure of hBuChE has been used (PDB ID: 4BDS). Docking calculations were performed following the same protocol described before for hAChE, not including flexibility at the receptor. All dockings were performed as blind dockings where a box of 66 × 66 × 70 Ǻ with grid points separated 1 Ǻ, was positioned at the middle of the protein (x = 136.0; y = 123.59; z = 38.56).

Default parameters were used except num_modes, which was set to 40. The docked conformations of each ligand were ranked based on the binding energy and the top ranked conformations were visually analyzed. The lowest docking-energy conformation was considered as the most stable orientation. Finally, the docking results generated were directly loaded into the Discovery Studio and interactions between docked ligands and macromolecule were analyzed.

## Figures and Tables

**Table 1:**
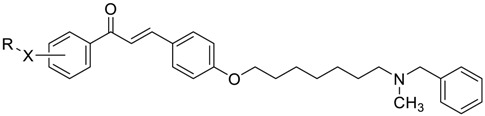
Inhibitory activities against *h*AChE and *h*BuChE, as IC_50_ values, of compounds **1**–**15** and reference compounds tacrine (THA) and galantamine (GAL).

Comp.	X	Chain Position	R	IC_50_ (µM) ± SEM		SI ^a^
				*h*AChE	*h*BuChE	
**1**	-	para	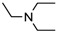	2.81 ± 0.29	8.83 ± 0.58	3.14
**2**	O	para	CH_3_	98.5 ± 8.8	2.85 ± 0.70	0.03
**3**	O	para	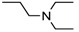	0.70 ± 0.09	19.9 ± 5.5	28.4
**4**	O	para	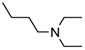	0.92 ± 0.07	2.38 ± 0.05	2.6
**5**	O	para	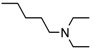	1.06 ± 0.08	3.70 ± 0.08	3.5
**6**	O	para	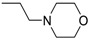	511 ± 37	11.0 ± 0.5	0.021
**7**	O	para	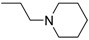	1.25 ± 0.06	19.7 ± 0.6	15.8
**8**	O	para	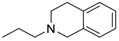	16.5 ± 2.6	3.87 ± 0.30	0.23
**9**	O	para	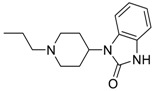	13.5 ± 2.3	3.93 ± 0.20	0.29
**10**	O	para	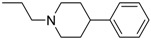	n.a. ^b^	n.a. ^b^	-
**11**	O	para	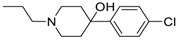	n.a. ^b^	n.a. ^b^	-
**12**	O	meta	CH_3_	105 ± 17	8.58 ± 0.35	0.08
**13**	O	meta	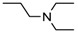	1.47 ± 0.11	0.92 ± 0.04	0.62
**14**	O	meta	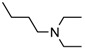	5.78 ± 0.67	1.82 ± 0.05	0.31
**15**	O	meta	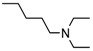	1.08 ± 0.17	2.37 ± 0.05	2.19
**THA**	-	-	-	0.32 ± 0.01	0.04 ± 0.00 ^c^	0.12
**GAL**	-	-	-	2.10 ± 0.15	20.7 ± 1.5	9.85

^a^ SI: selectivity index for AChE, calculated as IC_50_ *h*BuChE/*h*AChE ratio; ^b^ n.a. stands for “not active”, i.e., inhibition lower than 15% at the highest tested concentration; ^c^ IC_50_(BuChE) = 45.8 ± 0.3 nM.

**Table 2:**
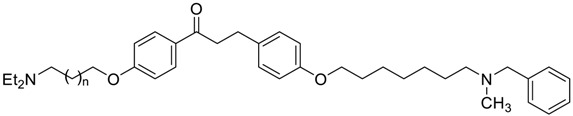
Inhibitory activities against *h*AChE and *h*BuChE, as IC_50_ values, of compounds **16**–**18**.

Comp.	*n*	IC_50_ (µM) ± SEM		SI ^a^
		*h*AChE	*h*BuChE	
**16**	0	6.21 ± 0.32	20.2 ± 1.7	3.25
**17**	1	1.66 ± 0.09	5.04 ± 0.30	3.04
**18**	2	2.46 ± 0.11	12.4 ± 2.4	5.04

^a^ SI: selectivity index for AChE, calculated as IC_50_ *h*BuChE/*h*AChE ratio.

## References

[1] Prince M., Wimo A., Guerchet M., Ali G.-C., Wu Y.-T., Prina M. (2015). World Alzheimer Disease Report.

[2] Winblad B., Amouyel P., Andrieu S., Ballard C., Brayne C., Brodaty H., Cedazo-Minguez A., Dubois B., Edvardsson D., Feldman H. (2016). Defeating Alzheimer’s disease and other dementias: A priority for European science and society. Lancet Neurol..

[3] Bachurin S.O., Bovina E.V., Ustyugov A. (2017). Drugs in Clinical Trials for Alzheimer’s Disease: The Major Trends. Med. Res. Rev..

[4] Contestabile A. (2011). The history of the cholinergic hypothesis. Behav. Brain Res..

[5] Ashraf G.M., Greig N.H., Khan T.A., Hassan I., Tabrez S., Shakil S., Sheikh I.A., Zaidi S.K., Akram M., Jabir N.R. (2014). Protein Misfolding and Aggregation in Alzheimer’s Disease and Type 2 Diabetes Mellitus. CNS Neurol. Disord. Drug Targets.

[6] Akiyama H., Barger S., Barnum S., Bradt B., Bauer J., Cole G.M., Cooper N.R., Eikelenboom P., Emmerling M., Fiebich B.L. (2000). Inflammation and Alzheimer’s disease. Neurobiol. Aging.

[7] Rosini M., Simoni E., Milelli A., Minarini A., Melchiorre C. (2014). Oxidative Stress in Alzheimer’s Disease: Are We Connecting the Dots?. J. Med. Chem..

[8] Hybertson B.M., Gao B., Bose S.K., McCord J.M. (2011). Oxidative stress in health and disease: The therapeutic potential of Nrf2 activation. Mol. Aspects Med..

[9] Rizzo S., Rivière C., Piazzi L., Bisi A., Gobbi S., Bartolini M., Andrisano V., Morroni F., Tarozzi A., Monti J.P. (2008). Benzofuran-based hybrid compounds for the inhibition of cholinesterase activity, β amyloid aggregation, and Aβ neurotoxicity. J. Med. Chem..

[10] Rizzo S., Tarozzi A., Bartolini M., Da Costa G., Bisi A., Gobbi S., Belluti F., Ligresti A., Allarà M., Monti J.-P. (2012). 2-Arylbenzofuran-based molecules as multipotent Alzheimer’s disease modifying agents. Eur. J. Med. Chem..

[11] Singh P., Anand A., Kumar V. (2014). Recent developments in biological activities of chalcones: A mini review. Eur. J. Med. Chem..

[12] Zhuang C., Zhang W., Sheng C., Zhang W., Xing C., Miao Z. (2017). Chalcone: A privileged structure in medicinal chemistry. Chem. Rev..

[13] Mendieta L., Picò A., Tarragò T., Teixidò M., Castillo M., Rafecas L., Moyano A., Giralt E. (2010). Novel Peptidyl Aryl Vinyl Sulfones as Highly Potent and Selective Inhibitors of Cathepsins L and B. ChemMedChem.

[14] Mertens M.D., Schmitz J., Horn M., Furtmann N., Bajorath J., Mares M., Gutschow M. (2014). A Coumarin-Labeled Vinyl Sulfone as Tripeptidomimetic Activity-Based Probe for Cysteine Cathepsins. ChemBioChem.

[15] Staskun B., van Es T. (2008). The Reduction of Nitriles to Aldehydes: Applications of Raney Nickel/Sodium Hypophosphite Monohydrate, of Raney Nickel/Formic Acid, or of Raney(Ni/Al)Alloy/Formic Acid, Respectively. S. Afr. J. Chem..

[16] Ellman G.L., Courtney K.D., Andres V., Featherstone R.M. (1961). A new rapid colorimetric determination of acetylcholinesterase activity. Biochem. Pharmacol..

[17] Nicolet Y., Lockridge O., Masson P., Fontecilla-Camps J.C., Nachon F. (2003). Crystal structure of human butyrylcholinesterase and of its complexes with substrate and products. J. Biol. Chem..

[18] Greig N.H., Lahiri D.K., Sambamurti K. (2002). Butyrylcholinesterase: An Important New Target in Alzheimer’s Disease Therapy. Int. Psychogeriatr..

[19] Weinstock M. (1999). Selectivity of cholinesterase inhibition: Clinical implications for the treatment of Alzheimer’s disease. CNS Drugs.

[20] Greig N.H., Utsuki T., Ingram D.K., Wang Y., Pepeu G., Scali C., Yu Q.-S., Mamczarz J., Holloway H.W., Giordano T. (2005). Selective butyrylcholinesterase inhibition elevates brain acetylcholine, augments learning and lowers Alzheimer β-amyloid peptide in rodent. Proc. Natl. Acad. Sci. USA.

[21] Darvesh S., Reid G.A. (2016). Reduced fibrillar β-amyloid in subcortical structures in a butyrylcholinesterase-knockout Alzheimer disease mouse model. Chem. Biol. Interact..

[22] Sengupta U., Nilson A.N., Kayed R. (2016). The Role of Amyloid-β Oligomers in Toxicity, Propagation, and Immunotherapy. EbioMedicine.

[23] Wu R., Hayashi T., Cottam H., Jin G., Yao S., Wu C., Rosenbach M., Corr M., Schwab R., Carson D. (2010). Nrf2 responses and the therapeutic selectivity of electrophilic compounds in chronic lymphocytic leukemia. Proc. Natl. Acad. Sci. USA.

[24] Satoh T., McKercher S.R., Lipton S.A. (2013). Nrf2/ARE-mediated antioxidant actions of pro-electrophilic drugs. Free Radic. Biol. Med..

[25] Hur W., Gray N.S. (2011). Small molecule modulators of antioxidant response pathway. Curr. Opin. Chem. Biol..

[26] Tarozzi A., Morroni F., Merlicco A., Hrelia S., Angeloni C., Cantelli-Forti G., Hrelia P. (2009). Sulforafane as an inducer of glutathione prevents oxidative stress-induced cell death in a dopaminergic-like neuroblastoma cell line. J. Neurochem..

[27] Tarozzi A., Morroni F., Bolondi C., Sita G., Hrelia P., Djemil A., Cantelli-Forti G. (2012). Neuroprotective effects of erucin against 6-hydroxydopamine-induced oxidative damage in a dopaminergic-like neuroblastoma cell line. Int. J. Mol. Sci..

[28] Morroni F., Sita G., Djemil A., D’Amico M., Pruccoli L., Cantelli-Forti G., Hrelia P., Tarozzi A. (2018). Comparison of Adaptive Neuroprotective Mechanisms of Sulforaphane and its Interconversion Product Erucin in in Vitro and in Vivo Models of Parkinson’s Disease. J. Agric. Food Chem..

[29] Tarozzi A., Bartolini M., Piazzi L., Valgimigli L., Amorati R., Bolondi C., Djemil A., Mancini F., Andrisano V., Rampa A. (2014). From the dual function lead AP2238 to AP2469, a multi-target-directed ligand for the treatment of Alzheimer’s disease. Pharmacol. Res. Perspect..

[30] Rampa A., Montanari S., Pruccoli L., Bartolini M., Falchi F., Feoli A., Cavalli A., Belluti F., Gobbi S., Tarozzi A. (2017). Chalcone-based carbamates for Alzheimer’s disease treatment. Future Med. Chem..

[31] Trott O., Olson A.J. (2010). AutoDock Vina: Improving the speed and accuracy of docking with a new scoring function, efficient optimization, and multithreading. J. Comput. Chem..

[32] Brooks B.R., Bruccoleri R.E., Olafson B.D., States D.J., Swaminathan S., Karplus M. (1983). CHARMM: A Program for Macromolecular Energy, Minimization, and Dynamics Calculations. J. Comput. Chem..

